# Behaviours that prompt primary school teachers to adopt and implement physically active learning: a meta synthesis of qualitative evidence

**DOI:** 10.1186/s12966-021-01221-9

**Published:** 2021-11-20

**Authors:** Andrew Daly-Smith, Jade L. Morris, Emma Norris, Toni L. Williams, Victoria Archbold, Jouni Kallio, Tuija H. Tammelin, Amika Singh, Jorge Mota, Jesper von Seelen, Caterina Pesce, Jo Salmon, Heather McKay, John Bartholomew, Geir Kare Resaland

**Affiliations:** 1 Center for Physically Active Learning, Faculty of Education, Arts and Sports, Western Norway University of Applied Sciences, Sogndal, Norway; 2 Faculty of Health Studies, University of Bradford, Bradford, UK; 3 Centre for Applied Education Research, Wolfson Centre for Applied Health Research, Bradford Royal Infirmary, Bradford, UK; 4 Centre for Society & Mental Health, Department of Health Services & Population Health, Institute of Psychiatry, Psychology & Neuroscience, King’s College London, London, UK; 5 Health Behaviour Change Research Group, Brunel University London, London, UK; 6 School of Sport, Carnegie, Leeds Beckett University, Leeds, UK; 7 Department of Sport and Exercise Sciences, Durham University, Durham, UK; 8 LIKES Research Centre for Physical Activity and Health, Jyväskylä, Finland; 9 Mulier Instituut, Utrecht, The Netherlands; 10 Research Center in Physical Activity, Health and Leisure, Faculty of Sport, University of Porto and Laboratory for Integrative and Translational Research in Population Health (ITR), Porto, Portugal; 11 Department for Research and Development, University College South Denmark, Haderslev, Denmark; 12 Department of Movement, Human and Health Sciences, University of Rome “Foro Italico”, Rome, Italy; 13 Institute for Physical Activity and Nutrition (IPAN), School of Exercise and Nutrition Sciences, Deakin University, Geelong, Australia; 14 Centre for Hip Health and Mobility, Vancouver Coastal Health Research Centre, Vancouver, Canada; 15 Department of Family Practice, University of British Columbia, Vancouver, Canada; 16 Department of Kinesiology and Health Education, The University of Texas at Austin, Austin, USA

**Keywords:** Systematic review, meta synthesis, Thematic synthesis, Physically active learning, School, Physical activity, Implementation, Theoretical domains framework, Behaviour, Teachers

## Abstract

**Background:**

Physically active learning (PAL) - integration of movement within delivery of academic content - is a core component of many whole-of-school physical activity approaches. Yet, PAL intervention methods and strategies vary and frequently are not sustained beyond formal programmes. To improve PAL training, a more comprehensive understanding of the behavioural and psychological processes that influence teachers’ adoption and implementation of PAL is required. To address this, we conducted a meta-synthesis to synthesise key stakeholders’ knowledge of facilitators and barriers to teachers’ implementing PAL in schools to improve teacher-focussed PAL interventions in primary (elementary) schools.

**Methodology:**

We conducted a meta-synthesis using a five-stage thematic synthesis approach to; develop a research purpose and aim, identify relevant articles, appraise studies for quality, develop descriptive themes and interpret and synthesise the literature. In the final stage, 14 domains from the Theoretical Domain Framework (TDF) were then aligned to the final analytical themes and subthemes.

**Results:**

We identified seven themes and 31 sub-themes from 25 eligible papers. Four themes summarised teacher-level factors: PAL benefits, teachers’ beliefs about own capabilities, PAL teacher training, PAL delivery. One theme encompassed teacher and school-level factors: resources. Two themes reflected school and external factors that influence teachers’ PAL behaviour: whole-school approach, external factors. Ten (of 14) TDF domains aligned with main themes and sub-themes: *Knowledge, Skills, Social/Professional Role and Identity, Beliefs about Capabilities, Beliefs about Consequences, Reinforcement, Goals, Environmental Context and Resources, Social influences* and *Emotio*n.

**Conclusions:**

Our synthesis illustrates the inherent complexity required to change and sustain teachers’ PAL behaviours. Initially, teachers must receive the training, resources and support to develop the capability to implement and adapt PAL. The PAL training programme should progress as teachers’ build their experience and capability; content should be ‘refreshed’ and become more challenging over time. Subsequently, it is imperative to engage all levels of the school community for PAL to be fully integrated into a broader school system. Adequate resources, strong leadership and governance, an engaged activated community and political will are necessary to achieve this, and may not currently exist in most schools.

**Supplementary Information:**

The online version contains supplementary material available at 10.1186/s12966-021-01221-9.

## Background

Schools are a privileged context to promote physical activity as they reach children from all social, cultural and economic groups [1, 2]. Many national policies emphasise the need for schools to provide opportunities for children to be active, with some countries mandating a required amount of physical activity [3–7]. Yet, despite policy support, there is much room for improvement as two recent meta analyses and a large pooled analysis of 20 controlled trials reported that most school-based approaches to physical activity are ineffective and lack sustainability [8–10].

Physically active learning (PAL) is considered a core component of many whole-school approaches to physical activity [11–13]. Specifically, PAL benefits physical activity during curricular lesson time and is defined as the integration of movement within delivery of academic content. The benefits of PAL to enhance physical activity and academic outcomes are outlined in recent meta-analyses and systematic reviews [14–16]. Importantly, benefits of PAL traverse all demographic subgroups [17]. Yet, PAL intervention methods and strategies vary and frequently are not sustained beyond the end of formal programs [18] .

Teachers are often primarily responsible for the adoption and implementation of PAL [19, 20]. However, few have received training and, therefore, lack the confidence to use PAL [21–23]. As a result, many teachers may hesitate to adopt PAL if it is viewed as complicated and an additional burden to a busy schedule [20, 24–26]. Hence, we require a better understanding of what teachers need to deliver PAL to inform the development of more comprehensive training programmes [21, 23, 27]. Through training, the needs of all teachers may be met, and the initial burden and time commitment of PAL are reduced [21, 28, 29]. Empowering teachers may also advance innovative, varied and more interesting approaches to PAL delivery [18, 30]. A recent systematic review on movement integration within curricular lessons further emphasised the need to identify the delivery attributes and teacher characteristics essential to facilitate PAL delivery [18]. Due to the rapid growth of qualitative PAL studies published over the last decade, the body of literature is prime for review in order to develop a comprehensive understanding the key factors that influence teachers PAL delivery.

We will use a meta-synthesis to systematically review and synthesise the qualitative literature to advance our understanding of essential PAL teacher behaviours [31]. Its aim is to search, appraise and synthesise findings from the PAL international literature into new themes that offer novel and more comprehensive insights into behaviours that promote teachers to implement PAL in schools [18]. Once synthesised, the findings can be used to create a framework of behavioural and psychological processes that will inform the future development of successful PAL teacher training programmes [32].

The Theoretical Domains Framework (TDF) of behaviour change synthesises key drivers of behaviour central to assess and inform intervention design and implementation [32, 33]. Derived from 33 cross-disciplinary theories and 128 key theoretical behavioural constructs, the TDF is comprised of 14 domains related to behaviour change; *Knowledge, Skills, Social/Professional Role and Identity, Beliefs about Capabilities, Optimism, Beliefs about Consequences, Reinforcement, Intentions, Goals, Memory, Attention and Decision Processes, Environmental Context and Resources, Social influences, Emotion* and *Behavioural Regulation* [33, 34]. Factors identified by the TDF show the important drivers impacting the uptake of a given behaviour, whereas the related COM-B model of behaviour change maps these TDF factors to develop corresponding interventions [31, 34]. The COM-B model presents that any behaviour will only occur if the person concerned has the c*apability* and *opportunity* to engage in the behaviour and the *motivation* to enact that behaviour rather than any other behaviours [34]. In this study, we will apply the 14 TDF domains in our meta-synthesis to present an in-depth understanding of the behaviours required by teachers to successfully adopt and implement PAL.

Specifically, the meta-synthesis will: (i) synthesise knowledge that conveys perceptions of key school stakeholder groups regarding facilitators and barriers to teachers’ implementing PAL in schools, and (ii) inform the systematic development of teacher-focussed interventions that enable PAL to be adopted, implemented and sustained in primary (elementary) schools.

## Methodology

The method is presented in five stages; stage one involved developing a research purpose and aims, stage two identification of relevant articles, stage three appraising the quality of included studies. Stages four and five involved developing descriptive themes through interpretation and conceptual synthesis drawing on the specific method of thematic synthesis [31, 35]. A thematic synthesis is a fluid, interpretive method that aims to address research questions that relate to intervention need, appropriateness, acceptability and effectiveness [36]. Using thematic synthesis shapes outputs that inform future policy, practice and research [31, 36]. Our interpretivist approach viewed primary qualitative research as a construction, and secondary research as a construction of a construction [37]. We recognise that our academic backgrounds and experience influenced how we interpreted our findings [38].

The study is a working package within the Erasmus+ funded ACTivate project (https://www.activateyourclass.eu/) that aims to develop a free-to-access online professional development package to improve PAL practice with teachers. The lead authors (ADS, JLM & GK) led the study on behalf of the ACTivate core team and the international advisory board. The author team has experience in PAL research and practice and have implemented and scaled up comprehensive school physical activity interventions in eight different countries. Throughout the thematic synthesis process, lead authors considered their reflexivity by examining their own beliefs, judgements and practices during the research process to identify how these may have influenced the interpretation of results. We accept that interpretation of findings from our meta-synthesis do not represent the only conclusions that could be drawn from the studies we reviewed. Results were constructed by authors at the time of completion, based on our skills and knowledge of the relevant literature, theories, practices and policies.

### Stage one: developing a research purpose and aims

First, the core team defined the purpose of the meta-synthesis and created clear research aims [31]. ADS and JLM drafted inclusion/exclusion criteria. Next, criteria were presented to the core team and IAB for feedback;

**Inclusion**

- Studies with participants that included anyone who has experienced delivery or training of a PAL programme in a primary (elementary) school setting. This included head teachers (principals), deputy heads (vice principals), senior management, teachers (including Physical Education teachers), teaching assistants, teacher trainers/ training organisations and university students.
- Studies that involved a qualitative review of an intervention or explorative study on stakeholder perceptions (e.g academic staff, public health specialists) of the integration of physical movement within the delivery of academic lessons (e.g numeracy, literacy).

#### Exclusion

- Studies conducted with stakeholders working on PAL interventions based in early years settings, secondary schools, colleges or universities
- Studies focussed on non-PAL interventions (e.g. classroom movement breaks without academic content)
- Quantitative research

After we finalised inclusion and exclusion criteria, we registered our review on PROSPERO (https://www.crd.york.ac.uk/prospero/; CRD42020202853).

### Stage two: identifying relevant articles

For the second stage we identified papers that used qualitative methods and were relevant to our research aims [31]. We adhered to PRISMA guidelines [39]. Electronic searches for articles were conducted on seven databases: (i) Academic Search Complete, (ii) ERIC, (iii) PubMed, (iv) PsychARTICLES, (v) PsychINFO, (vi) SCOPUS and (vii) SPORTDiscus. The search strategy contained three strings with multiple terms within each string (Supplementary material A). The three strings included search terms for: (i) PAL or movement strategies, (ii) stakeholders and (iii) facilitators, barriers and implementation of PAL. The search was conducted in December 2020, with no date limiters. Three existing systematic reviews on PAL were identified [18, 40, 41] and searched for additional articles that may align to our screening process. The ACTivate core team and IAB were asked to identify relevant authors and papers to ensure a comprehensive review of the relevant literature. Following completion of the searches, ADS and JLM dual screened all papers using the inclusion/ exclusion criteria. First, articles were screened by title and abstract. Next, we conducted a full text screening of eligible studies.

### Stage three: appraising studies for quality

For the third stage we appraised the research quality of eligible articles [31]. Quality appraisal comprises of judging the theoretical, methodological and/or analytical components of individual studies to avoid drawing any unreliable or misleading conclusions and recommendations [36, 42]. There are no agreed methods for judging individual study quality within the syntheses of qualitative evidence [42, 43]. Making judgements over the trustworthiness and rigor of qualitative research is challenging [44]. We applied Garside’s [45] quality appraisal recommendations for qualitative synthesis as per previous meta-synthesis [46]. To do so we considered three primary constructs of all included studies: (i) trustworthiness (considering epistemological aspects), (ii) theoretical considerations, and (iii) practical considerations (technical aspects). Two authors (ADS and JLM) assessed all three constructs of study quality. Trustworthiness focused on the appropriateness of the design and execution to answer the research question(s). Theoretical considerations were judged on whether studies included theoretical frameworks or models, and if conclusions were supported by the data. Practical considerations were assessed based on the contribution of the study to themes and sub-themes. The detail of the methods and frameworks used within each individual study is presented within Table 1.

**Table 1:** Summary of the articles included within the thematic synthesis

Study	Aim	Sample	Study design	Theoretical framework/model	Data collection	Analysis
Benes et al. (2016) USA [47]	Examine classroom teachers’ perceptions about integrating movement in the classroom.	Teachers (*n* = 17, 15 females).	Cross-sectional: assessing classroom-based movement integration.		Semi-structured interviews.	Drew upon grounded theory. General inductive analysis
Daly-Smith et al. (2020) England, UK [13]	To identify multi-stakeholder perspectives deemed important for successful widespread PAL implementation and adoption.	Researchers (*n* = 15), policy/local authority (*n* = 9), teachers (*n* = 3) and commercial education sector (*n* = 8)	Cross-sectional: multi-stakeholder PAL implementation.	New framework based on socio-economic model proposed within the discussion.	Solution-based workshops with five heterogenous and multi-disciplinary groups.	Open coding analysis
Dorling et al. (2020) UK [48]	Assess underlying mechanisms relating to stakeholders and the effectiveness of practices demonstrated by EduMove student practitioners.	Teachers (*n* = 5) and student practitioners (*n* = 6).	Cross-sectional: assessing participants’ experiences of the EduMove programme.	The COM-B model was used to interpret results	Semi-structured interviews.	Thematic analysis
Dugger et al. (2020) USA [49]	Examine elementary classroom teachers’ self-reported use of different MI products and identify teachers’ perceived facilitators and barriers.	Teachers (*n* = 40)	Intervention: Testing four movement integration products for five days.		Focus groups	Drew upon grounded theory and immersion crystallization. Inductive analyses using latent coding techniques.
Dyrstad et al. (2018) Norway [50]	Understand school leaders’, teachers´ and children’s responses to the PAL lessons and facilitators and barriers to implementing PAL lessons?	Teachers (*n* = 13), principals (*n* = 3), vice-principals (*n* = 2), and children (*n* = 6)	Process evaluation: PAL implementation embedded in the ‘Active school’ RCT.	Data interpretation was inspired by Fullan’s (2007) theoretical framework	Teacher focus groups and school leader interviews 8 weeks into the intervention and post interventions. Post intervention focus groups with children.	Qualitative content analysis.
Egan et al. (2018) USA [51]	To qualitatively examine the program implementation process from the perspective of the teachers who taught in the intervention classrooms.	Teachers (*n* = 9)	Intervention: Year one of a pilot PACES non-RCT.	The intervention was based on a partnership model (Webster et al., 2015)	Semi-structured interviews	A narrative inquiry methodology used to code interviews.
Gately et al. (2013) England, UK [52]	Explore teachers’ perspectives of the implementation of the TAKE 10! programme.	Teachers (*n* = 8).	Intervention: One school year embedding TAKE 10!		Semi-structured interviews at three time-points.	Thematic analyses
Gibson et al. (2008) USA [53]	Understand teachers’ perceptions about PAAC and the challenges and barriers to achieving 90 min of active lessons per week.	Teachers (*n* = 79).	Intervention: PAAC is a cluster-RCT, elementary school-based 3-year trial.	Process evaluation components guided by Linnan and Steckler (2002); Baranowski and Stables (2000)	Teacher focus groups.	Content analysis techniques
Goh et al. (2017) USA [54]	What are facilitators and barriers in the implementation of TAKE 10! and what are key factors associated with teachers continued use of TAKE 10!?	Teachers (*n* = 15, 11 females).	Intervention: 8-weeks of TAKE 10!	Comprehensive School Physical Activity Program Model	Semi-structured interviews post intervention.	Open-coding methodology
Graham et al. (2014) USA [24]	Understand the current PA climate, school and school personnel readiness to change, and perceived benefits and barriers to increased PA and the hypothetical use of *JumpIn!*.	Teachers (*n* = 11) and principal (*n* = 1). 8% males	Cross-sectional		Six focus groups were conducted using a semi-structured interview guide.	A theme-based approach was used to analyse the results.
Kain et al. (2020) Chile [55]	Explore the barriers and facilitators to implementation of the developed PAL materials, and pilot test effectiveness.	Teachers (*n* = 14, all female).	Intervention: PAL implementation for 73 days.		Teacher reported implementation logs followed by semi structured interviews.	Drew on grounded theory. Content analysis.
Lander et al. (2020) Australia [20]	Investigate the reach, effectiveness, adoption, adaption, implementation and maintenance of Transform-Ed! across the first year of an undergraduate teacher course.	Senior academics (*n* = 5), lecturers (*n* = 6) and undergraduate, pre-service teachers (*n* = 274)	Intervention: 12-week Transform-Ed! programme, embedded into a core curriculum.	The design, implementation, and evaluation was guided by the RE-AIM framework.	Semi-structured interviews (with senior academics and lecturers) and focus groups (students).	Coding aligned to the RE-AIM framework. .
Lerum et al. (2019) Norway [13]	Describe teachers’ experiences of implementing the ASK intervention and investigate teachers’ maintenance of the ASK intervention.	Teachers (*n* = 26).	Cross-sectional: Follow-up with teachers involved in the ASK intervention.	The intervention was co-produced with teachers and other school stakeholders using the COM-B model	Self-report questionnaire. With open-ended questions at two time points.	Thematic analysis
Marchant et al. (2019) Wales, UK [21]	Examine acceptability and explore headteachers, teachers and pupils’ views and experiences of outdoor learning within the key stage two curriculum.	Headteachers (*n* = 3), teachers (baseline: *n* = 4, follow up: *n* = 6) and pupils (baseline: *n* = 4, follow up: *n* = 6).	Intervention: Six-month outdoor learning programme, one outdoor lesson a week.		Focus groups (pupils), interviews (teachers and headteachers).	Thematic analysis
McMullen et al. 2016) Ireland [25]	Engage teachers’ voices in order to determine factors that encourage and inhibit their adoption of academically linked movement integration practices in their classrooms.	Teachers (*n* = 13, all female).	Intervention: One school part of a larger pilot study implementing Moving to Learn Ireland.	Comprehensive School Physical Activity Program Model	Pre- and post- questionnaires, structured teacher lesson reflections, focus group interviews, and field notes generated from workshops.	Inductive coding using interpretive approach.
Mwaanga et al. (2018) Isle of Wight, UK [56]	How is PAL pragmatically embedded and managed within classrooms.	Primary school teachers (*n* = 7), programme coordinator (*n* = 1).		Socio-ecological model	Realist interviews were conducted with all participants to gather initial exploratory data.	
Norris et al. (2015) England, UK [57]	Assess current PAL practices, and teacher and pupil attitudes towards physically active virtual field trips (VFT).	Teachers (*n* = 12) and pupils (*n* = 18).	Intervention: Part of a larger-scale assessment on school VFT engagement.	Technology Acceptance Model used to interpret teachers’ acceptability to VFT in the discussion.	Teacher semi-structured interviews and pupil focus groups.	Thematic analysis
Norris et al. (2018) England, UK [58]	Evaluate the processes underlying the Virtual Traveller intervention according to RE-AIM framework criteria.	Pupils (*n* = 6, 2 from lower, middle and higher overall academic ability).	Intervention: 6-weeks.	Findings are reported according to the RE-AIM	Semi-structured focus group.	Thematic analysis
Quarmby et al. (2018) [23] England, UK	Explore primary school teachers’ perceptions of PAL and map out barriers to a socio-ecological model.	Practising teachers (*n* = 31, 23 female) from 9 different primary schools	Cross-sectional.	Socio-ecological model	Six semi-structured focus group interviews	Thematic analysis
Riley et al. (2017) Australia [30]	Explore students’ and teachers’ perceptions of a maths-based PAL.	Students (*n* = 66, 50% female) and teachers (*n* = 4)	Intervention: EASY Minds, a cluster RCT delivered for 6-weeks.	The NWS Quality Teaching Model was used for teacher training and to inform the results and discussion.	11 semi-structured student focus groups and teacher interviews.	General inductive approach
Routen et al. (2018) England, UK [26]	Explore UK primary school class teacher’s views on MI, identifying perceived factors associated with delivery and implementation.	Teachers (*n* = 19), teaching assistants (*n* = 6). 21 were female.	Cross-sectional.	Socio-ecological model	Semi-structured face-to-face interviews were primarily used.	Thematic analysis
Skage et al. (2020) Norway [59]	What were the teachers’ use of PAL at two-year follow-up? and what are the factors affecting continued use of PAL?	School leaders (*n* = 5) and teachers (*n* = 9, 6 females).	Intervention: Two-year follow up of a 10-month cluster RCT PAL.	Concerns Based Adoption Model was used as a conceptual framework.	Semi-structured individual interviews with teachers and school leaders.	Thematic analysis
Skage & Dyrstad (2019) Norway [60]	Explore head teachers’ perceptions of PAL to identify factors affecting headteachers’ approval or rejection of PAL implementation.	Headteachers in primary and secondary schools (*n* = 29, 62% female).	Cross-sectional.	Aligned discussion briefly with Quality Implementation Framework	Semi-structured telephone interviews.	Content analysis
Stylianou et al. (2016) USA [61]	Examine teachers’ self-reported practices and perceptions of classroom-based PA including the training and implementation process.	Teachers (*n* = 13, 12 female).	Intervention: a comprehensive school health and PA project.	Drew upon Guskey’s (2002) alternative model of teacher change within the method, results and discussion.	Teacher self-reported on implementation and reflections, teacher observations and semi-structured interviews.	Constant comparison and analytic induction techniques
Webster et al. (2017) USA [62]	Examine teachers’ perspectives on MI while participating in a school-based pilot program.	Teachers (*n* = 12, 10 females).	Intervention: Part of the PACEs programme.	Interview questions were based on theoretical basis (theory of planned behaviour, social ecological model; social learning theory; teacher socialization theory; diffusion of innovations theory) and drawn upon in the discussion.	Semi-structured interviews.	Used grounded theory and immersion crystallization procedures. Latent coding technique.

Note. *COM-B* capabilities, opportunity and motivation = behaviour; *CBPA* classroom-based physical activity; *MI* movement integration; *PA* physical activity; *PAL* physically active learning; *RE-AIM* (reach, effectiveness, adoption, implementation, and maintenance); *RCT* randomised controlled trial; *VFTs* virtual field trips. For studies that encompassed a mixed-methods approach, only the qualitative assessment, relevant to our meta-synthesis has been included within this table.

First, for trustworthiness, we considered whether each article was designed and executed appropriately, answered research questions and provided conclusions supported by data. Some studies included strategies to increase trustworthiness of the data collection process and credibility of the data (see Table 1, column data analyses). One example is drawing on triangulation methods [23, 25, 26, 47, 51, 54, 60]. Second, for theoretical frameworks or models, most studies connected to a wider body of knowledge throughout, often drawing together the current evidence base in conjunction with the study findings. In one instance, a new framework was proposed within the study discussion [19]. Some studies paid particular attention to the Comprehensive School Physical Activity Programme (CSPAP) model [25, 54], where others drew upon the socio-ecological model [23, 26, 56]. While not all studies explicitly drew upon theoretical frameworks/ models, this did not influence the usability of the results for our synthesis. Third, for practical considerations, we accepted that all studies contributed to this review (see Table 2 for study contribution against each sub-theme).

**Table 2:** Mapping the alignment of papers, themes, subthemes and Theoretical Domains Framework domains

Theme	Sub-theme		Theoretical Domains Framework domains	Papers
PAL Benefits	Teachers’ motivation and perceived effects		Knowledge (#1); beliefs about consequences (#6), reinforcement (#7); goals (#9)	1, 2, 5, 7, 8, 9, 13, 14, 15, 19, 20, 21, 23, 24, 25
	Embracing class diversity		Knowledge (#1); beliefs about consequences (#6); goals (#9)	7, 14, 15, 19, 20, 23
	Lack of dissemination of evidence/ communication disparities		Knowledge (#1); beliefs about consequences (#6); social influences (#12)	1, 2, 13
	Pupil’s educational outcomes		Knowledge (#1); beliefs about consequences (#6)	1, 2, 3, 4, 5, 7, 8, 10, 12, 13, 14, 15, 16, 18, 19, 20, 21, 22, 23, 24, 25
	Pupil’s health		Knowledge (#1); beliefs about consequences (#6); emotion (#13)	2, 3, 4, 5, 7, 14, 15, 16, 17, 18, 19, 20, 21, 23, 24, 25
	Pupil’s social engagement and teamwork		Knowledge (#1); beliefs about consequences (#6)	1, 13, 14, 18, 20
	Pupil’s enjoyment and motivation		Knowledge (#1); beliefs about consequences (#6); emotion (#13)	1, 3, 4, 5, 7, 9, 13, 14, 15, 16, 18, 20, 22, 24, 25
	Classroom behaviour		Knowledge (#1); beliefs about consequences (#6)	1, 3, 4, 5, 7, 13, 14, 18, 20, 21, 23, 24
Teachers’ beliefs about own capabilities	Attitudes towards PAL		Belief about capabilities (#4)	11, 12, 15, 18, 19, 22, 23, 25
	Confidence in using PAL		Skills (#2); belief about capabilities (#4)	3, 11, 16, 17, 18, 19, 21
	Trial and error		Skills (#2); belief about capabilities (#4)	1, 5, 9, 10, 13, 14, 15, 16, 19, 25
	Idea generation		Skills (#2); social/ professional role and identify (#3); belief about capabilities (#4)	1, 3, 8
PAL teacher training	Importance of PAL training		Skills (#2); social/ professional role and identify (#3); belief about consequences (#4); reinforcement (#7)	2, 9, 14, 16, 18, 20, 25
	Awareness and knowledge of PAL		Knowledge (#1)	1, 2, 10, 13, 17, 25
	PAL examples, demonstrations and direct experiences		Skills (#2); social/ professional role and identify (#3); belief about consequences (#6).	2, 5, 6, 8, 9, 13, 18, 20, 21, 24
	Tailored ongoing support		Skills (#2), goals (#9); social influences (#12)	6, 13, 15, 19, 21, 24
PAL delivery	Planning (lesson integration)		Skills (#2)	2, 3, 4, 6, 9, 14, 15, 18, 22, 24, 25
	Frequency		Knowledge (#1); Beliefs about consequences (#6)	1, 14, 15, 23
	Intensity		Skills (#2); Beliefs about consequences (#6)	2, 5, 14, 15, 17
	Subject compatibility		Skills (#2)	2, 10, 17, 18, 19, 25
	Differentiation	Academic	Skills (#2); beliefs about consequences (#6)	1, 5, 7, 9, 10, 11, 21, 24, 25
		Physical	Skills (#2)	9, 10, 19
		Psychosocial	Skills (#2); beliefs about capabilities (#4); emotion (#13)	1, 9, 14, 21, 25
		Age	Skills (#2); beliefs about consequences (#6)	3, 4, 24, 25
Resources	Time		Environmental context and resources (#11)	1, 2, 3, 4, 5, 6, 7, 8, 9, 10, 13, 14, 15, 16, 20, 21, 22, 23, 24, 25
	PAL delivery resources		Environmental context and resources (#11)	2, 4, 5, 9, 10, 12, 13, 14, 15, 16, 18, 19, 21, 24, 25
	Delivery environments		Skills (#2); environmental context and resources (#11)	1, 2, 4, 5, 6, 7, 8, 9, 10, 11, 14, 15, 16, 18, 19, 21, 25
	School finance		Environmental context and resources (#11)	14, 20, 23
Whole-of-school approach	The role of school culture in implementing PAL		Social/professional role & identity (#3); environmental context and resources (#11); social influences (#12)	1, 2, 3, 15, 21, 22, 25
	Sustainable implementation of PAL dependent on whole-of-school approach		Environmental context and resources (#11); social influences (#12)	2, 5, 11, 12, 14, 16, 20, 21, 22
	Senior leaders support for PAL culture		Environmental context and resources (#11); social influences (#12)	2, 5, 10, 13, 14, 16, 19, 21, 23, 24, 25
	Teamwork and collaboration		Social/ Professional Role and Identity (#3); Social influences (#12)	5, 6, 8, 9, 12, 13, 14, 21, 25
External factors	Policy (education & health)		Reinforcement (#7); environmental context and resources (#11)	2, 12, 14, 15, 19, 21, 23
	Parents		Environmental context and resources (#11); social influences (#12)	14, 19, 21, 25

1 Benes et al., 2016; 2 Daly-Smith et al., 2020; 3 Dorling et al., 2020; 4 Dugger et al., 2020; 5 Dyrstad et al., 2018; 6 Egan et al., 2018; 7 Gately et al., 2013; 8 Gibson et al., 2008; 9 Goh et al., 2017; 10 Graham et al., 2014; 11 Kain et al., 2020; 12 Lander et al., 2020; 13 Lerum et al., 2019; 14 Marchant et al., 2019; 15 McMullen et al., 2016; 16 Mwaanga et al., 2018; 17 Norris et al., 2018; 18 Norris et al., 2015; 19 Quarmby et al., 2018; 20 Riley et al., 2017; 21 Routen et al., 2018; 22 Skage et al., 2020; 23 Skage & Dyrstad 2019; 24 Stylianou et al., 2016; 25 Webster et al., 2017

### Stage four: developing descriptive themes

For stage four we extracted the data, coded the text and developed descriptive themes [36]. All text included in ‘results’, ‘findings’ and ‘discussion’ sections was extracted from eligible studies. This included any tables or figures that presented participant quotes and/or authors’ interpretations of the data. Any text included in the results and/or discussion where the authors were referring to additional literature in the field (e.g., secondary citations) was excluded. Where studies included results that encompassed non-PAL interventions (e.g., classroom movement breaks) this data was not extracted to not contaminate the focus on PAL of this review with potentially spurious effects. However, when it was not possible to distinguish between the type of physical activity, this data was included within the analysis. Next, data were coded to allow us to translate key concepts across articles [36]. We used inductive coding on all extracted data. That is, we coded one line at a time with no limitations on the style or interpretation of the coding. Inductive coding ensured that new concepts were not omitted which may occur if coding deductively in line with a prior framework [36, 46].

Lead authors (ADS and JLM) first extracted and coded one article simultaneously and discussed their analysis of data, before coding all articles. In line with our interpretivist approach, this reflexive exercise explores alternative interpretations and explanations of the data [46], rather than to seek reliable coding and claim inter-rater reliability [44]. Next, the same authors independently extracted and coded the remaining articles, reviewing coding outcomes together as the analysis progressed. Finally, similarities and differences in the codes were discussed prior to the iterative development of the data-driven descriptive themes [36].

### Stage five: interpretation and conceptual synthesis

For stage five, we constructed analytical themes through interpretation and conceptual synthesis [36]. We moved from data-driven descriptive themes to constructing theory-driven analytical themes where new or enhanced knowledge emerged. To construct themes underpinned by theory, descriptive themes were interrogated against the current literature, theoretical frameworks and our review aims. During this stage, we deductively drew upon the TDF to further refine our themes and subthemes while ensuring the language used remained aligned with the educational context. The TDF construct was only identified if the text explicitly mentioned characteristics of the domain. We applied this understanding across all analyses. TDF constructs were also only included if they aligned with the behaviour of the teacher, not that of the pupils as is the case for the decision processes. The ACTivate core team engaged in two workshops to enhance this process. The lead authors presented descriptive themes and gathered insights from the ACTivate team to re-define, re-shape and enhance ‘theory-driven’ themes. Following this, a final workshop was held with the IAB. Through this process the wider team of ‘experts’ in PAL research scrutinised themes and offered additional and alternative insights into key findings. Main theme and sub-theme names were aligned with the educational context.

Next, ADS, JLM and EN aligned the final themes and sub-themes with the fourteen TDF constructs. First, they individually mapped TDF constructs to themes and sub-themes via interpretation and alignment of the TDF construct with the identified quotes. Next, they met three times to discuss and agree upon alignment. All three authors accepted that more than one TDF domain may apply to a theme or sub-theme. We discuss each theme in the results section, paired with relevant concepts from the TDF with an accompanying narrative.

## Results

**Fig. 1:**
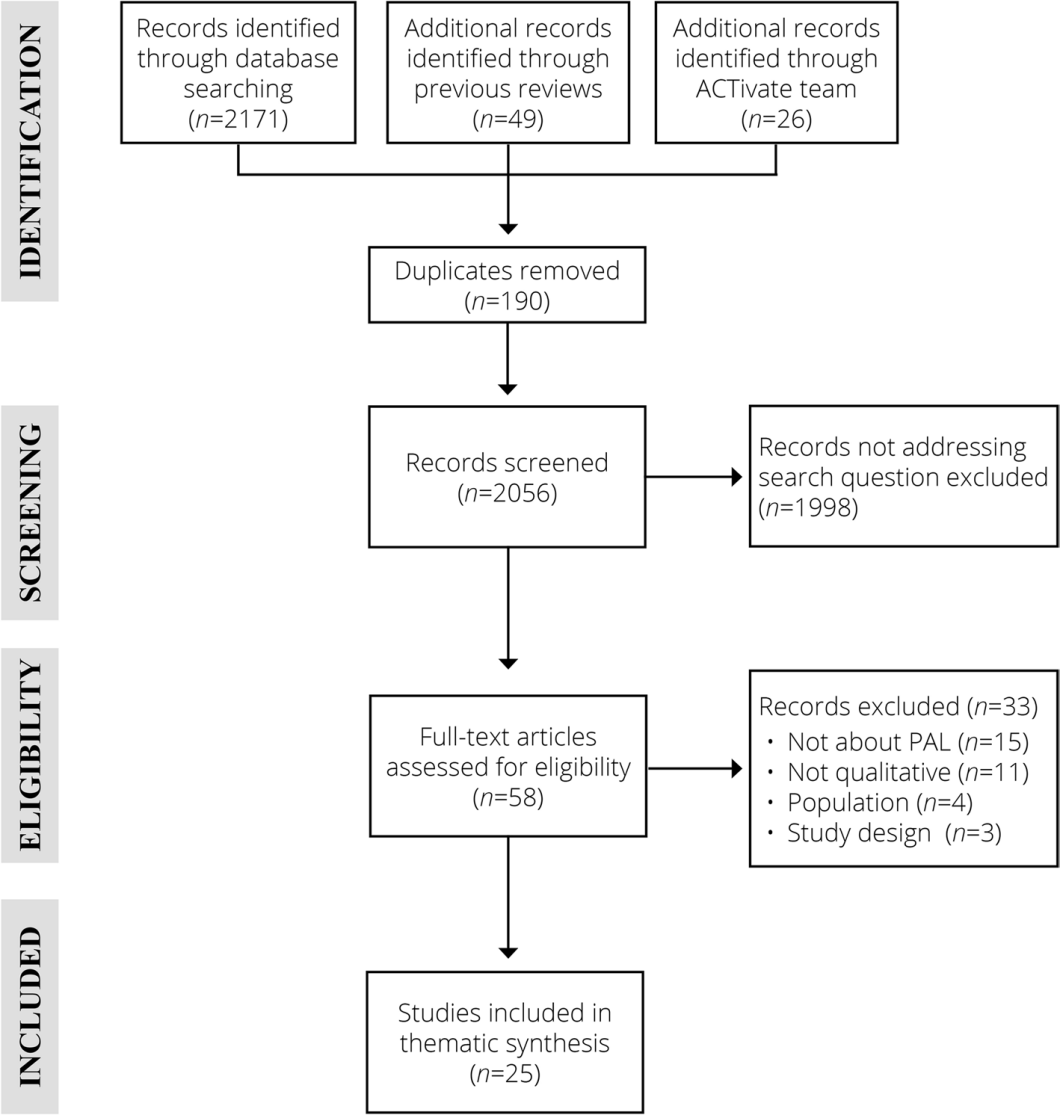
PRISMA flow chart illustrating study inclusions through the stages of the meta synthesis. PAL, physically active learning; PRISMA, Preferred Reporting Items for Systematic Reviews and Meta-Analyses

**Fig. 2:**
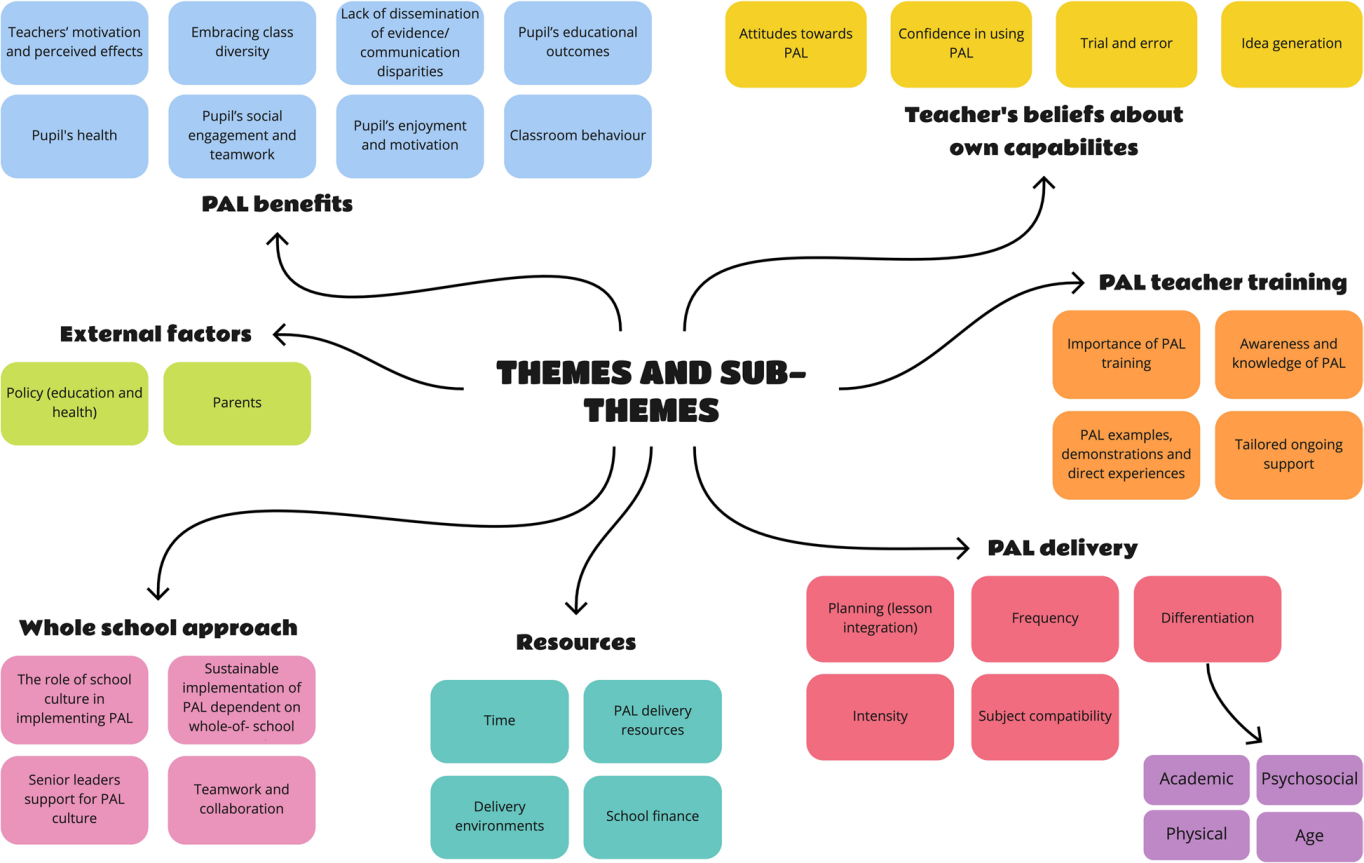
Map of themes and sub-themes that underpin teacher adoption and implementation of PAL

We identified seven overarching themes from the analysis of 25 eligible papers (Fig. 1, Table 1). Themes were: (1) PAL benefits, (2) Teachers’ beliefs about own capabilities, (3) PAL teacher training, (4) PAL delivery, (5) resources, (6) whole-school approach, (7) and external factors (Table 2). Overarching themes provide a summary of teachers’ perspectives on facilitators and barriers to implementing PAL in primary (elementary) schools. Themes one to four summarise teacher-level factors, theme five includes teacher and school-level factors, while themes six and seven reflect school and external factors that influence a teacher’s PAL behaviour. A thematic map (Fig. 2) presents the links between the themes and sub themes. Ten (of 14) TDF domains aligned with main themes and sub-themes: *Knowledge, Skills, Social/Professional Role and Identity, Beliefs about Capabilities, Beliefs about Consequences, Reinforcement, Goals, Environmental Context and Resources, Social influences* and *Emotio*n (Table 2). The four TDF themes that were not identified within the analysis were: *Optimism, Intentions, Behavioural Regulation* and *Memory, Attention and Decision Processes.* A table with a more comprehensive list of the extracted material can be found in Supplementary material, Table 1.

### PAL benefits

Eight sub-themes emerged; three aligned with how benefits play a role in whether teachers adopt and implement PAL. Five themes were related to pupil-level benefits. The three teacher-level sub-themes were; (i) teachers’ motivation and perceived effects, (ii) embracing class diversity, and (iii) lack of dissemination of evidence/communication disparities. *Knowledge* and *Beliefs about Consequences* were TDF domains related to all teacher sub-themes. *Reinforcement*, *Goals* and *Social Influences* were also identified. Many teachers reflected that seeing the benefits of PAL on pupils increased their motivation levels to continue to facilitate PAL-based lessons [30, 61]. Further, facilitating PAL lessons improved teachers’ understanding of the diverse needs of their class [21–23, 30, 52, 60]. This was due to PAL enabling different pupils to thrive and actively contribute within learning activities [60]. By providing an opportunity for children to express themselves physically and verbally, especially outdoors, PAL improved creativity and enhanced engagement [21]. PAL facilitated teachers’ self-reflection on their teaching styles, specifically related to creativity and levels of engagement [21]. Finally, teachers noted that evidence to support PAL’s effectiveness had not been disseminated into the practice literature [19, 20, 47]. Publishing articles in practitioner-oriented publications (websites, magazines), was thought to have greater likelihood of increasing teacher’s awareness of PAL benefits and support PAL uptake as part of ‘normal’ school practice.

At the pupil-level, the remaining five sub-themes were (iv) educational outcomes, (v) health, (vi) social engagement and teamwork, (vii) enjoyment and motivation, and (viii) classroom behaviour. These themes influence teachers’ motivation to deliver PAL [63]. *Knowledge*, *Beliefs about Consequences and Emotion* were TDF domains related to pupil sub-themes. Several studies suggested PAL presents alternate and beneficial learning approaches for pupils [19, 30, 61]. In some instances, PAL increased students’ understanding of the academic content [30]. Teachers felt PAL benefitted pupils’ daily PA levels, fitness levels and motor skills; greatest impact was on children with poor motor development or low aerobic fitness levels [13, 21, 23, 25, 26, 30, 48–50, 52, 56–58, 60–62]. While pupils perceived that PAL lessons could be physically challenging, they enjoyed movement opportunities [52]. Teachers and pupils perceived that PAL had benefits beyond core academic outcomes; that PAL enhanced social, teamwork and communication skills [20, 21, 30, 47, 57]. In addition, teachers noted that PAL lessons improved pupil enjoyment and motivation. This, in turn, increased engagement with academic content. PAL was used by many teachers to improve engagement. They used *‘active learning breaks’* to enable pupils to refocus for the subsequent lesson [26, 52, 57]. However, some teachers felt PAL increased behavioural problems [26]. Yet, this was mainly due to the novelty of PAL and teachers’ inexperience delivering PAL lessons in new environments [26].

### Teachers’ beliefs about own capabilities

Underpinning PAL adoption and implementation by teachers, are teachers’ belief in their capability. This was captured by four sub-themes: (i) attitudes towards PAL, (ii) confidence in using PAL, (iii) trial and error and (iv) idea generation. The TDF domain, *Beliefs about Capabilities,* was related to all sub-themes. *Skills* and *Social/Professional Role and Identity* were also identified. Teachers and schools were motivated to engage in PAL programmes, and through reflection noted the positive role that movement can play in a child’s day [25, 60]. However, some studies reflected a reticence of teachers to engage, as they were tired of change [60]. A lack of belief in the importance of PAL coupled with inadequate skills and knowledge to implement PAL effectively, might explain teachers’ reluctance to engage [62].

Teachers’ confidence was essential to improve PAL implementation. Confidence was underpinned by a teachers’ capacity to develop PAL delivery and manage pupil behaviour [26, 56]. That *‘every teacher’s nightmare is, oh my god, I’ve lost control [of the class]*’ reflects how teachers’ confidence to deliver PAL was directly linked to their capability to manage pupil behaviour [26]. To build belief in their own competence, it was essential teachers embraced a trial-and-error approach and *‘how to use movement in the classroom’* [47]. A sense of *‘fumbling through’* and *‘figuring it out’* with other teachers was common in the early stages of PAL delivery. Elevated confidence generated a willingness to try new ideas in future classes [54]. Finally, teachers whose confidence grew reported finding lessons easier to prepare [50]. Supporting teachers to be creative and generate their own ideas may enhance teachers’ confidence [47, 48]. Demonstrations by others were also used to generate new ideas [53].

### PAL teacher training

We identified four sub-themes within the *training* theme: (i) importance of PAL training, (ii) awareness and knowledge of PAL, (iii) PAL examples, demonstrations and direct experiences, and (iv) tailored ongoing support. TDF domains common to these sub-themes were *Skills* and *Social/Professional Role and Identity*, *Knowledge, Beliefs about Consequences, Reinforcement, Goals* and *Social Influence*s.

There was a lack of PAL training within initial teacher training and future courses that embed PAL training were suggested [13]. Lack of training was a key barrier to PAL implementation [56]. However, teachers who received PAL training found it highly engaging. Even with only minimal training their PAL practice improved [30, 54]. Without training, many teachers perceived PAL as a break from learning, rather than movement integrated into learning. Training programmes were deemed essential for teachers to realise the broad benefits of PAL [20, 24]. Seeing PAL in action or teachers trying it for themselves accelerated learning [61]. Teachers found sessions on the *‘use [of] different equipment [and] how to variate PAL lessons’* very useful [20]. Finally, tailored and ongoing support that reinforced learning was essential to facilitate teachers’ implementing PAL. Some teachers referred to *‘a sticky note for the mind’* to help reinforce actions [51].

### PAL delivery

Five sub-themes were aligned with PAL delivery: (i) planning (lesson integration), (ii) frequency, (iii) intensity, (iv) subject compatibility, and (v) differentiation for pupils’ needs. TDF domains common in these sub-themes were *Skills* and *Beliefs about Consequences, Knowledge, Beliefs about Capabilities* and *Emotion*.

Teachers were concerned about planning so as to fully integrate PAL lessons into the timetable. Teachers felt that to optimise the success of PAL, it needed to be fully integrated into lessons and *‘that it wasn’t something just separate, like that they were part of the lesson’* [25]. *‘High implementers indicated they were able to recognise and capitalise on naturally occurring transition times to integrate new movement opportunities.’* [51]; this quote suggests that planning ahead was not always necessary. Expectations as to how frequently PAL should be delivered varied between one and six lessons per week [21, 25], but it was important for teachers to use their own initiative [60].

While teachers need to deliver PAL at an intensity that yields health benefits, *‘it may not be feasible for schools to focus on meeting intensity targets when starting to implement PAL’* [19]. The intensity of the PAL lesson would depend on many factors, including the environment and subject matter [64]. *‘Resource cards in a box could mark activities as light, moderate or vigorous’* to support teachers to promote health-enhancing moderate-to-vigorous intensity [25, 64]. Compatibility allows teachers to use PAL in flexible ways and align it to different learning activities and curricular subjects. However, PAL was sometimes deemed inappropriate due to learning task requirements [23].

It was important for teachers’ to be able to adapt lessons to meet the different academic, physical and psychosocial needs of pupils. From an academic perspective, the physicality of PAL provides an alternate approach that can enhance learning or help reinforce learning by associating lessons with a fun activity [52]. However, repeating the same activities too often might precipitate a stale learning environment and hinder progression [50]. Needs of pupils differed by age and maturity. Teachers, therefore, needed the capability to adapt lessons based on age [48, 49]. The ability of teachers to deliver an inclusive PAL lesson that met the physical needs of all pupils was a concern for some. To illustrate, *‘from my son’s point of view, he’s a wheelchair user, that when they do that sort of thing in high school, he’s left at the side. Or because it takes him so much time to get into groups of organisation, that he always ends up with that person that no one else wants to work with. So, it’s about ensuring those sorts of physical aspects don’t isolate people’* [23]. Finally, teachers needed to be sensitive to the psycho-social needs of a diverse range of pupils and alter the delivery environment and activities to meet those needs [21]. To illustrate, pupils raised concerns about perspiring and not wanting ‘to look silly’ [62].

### Resources

Resources were deemed an integral element to high-quality PAL delivery. They aligned with the environment and resources component of the TDF. While availability of resources was often beyond teachers’ control, teachers’ ability to make the best use of resources directly influenced the quality of PAL. Four resources sub-themes emerged: (i) time, (ii) PAL delivery resources, (iii) delivery environments, and (iv) school finance. *Environmental Context and Resources* TDF domain was common to all sub-themes. *Skills* aligned to the effective delivery environments sub-theme.

Two-thirds of the studies identified time as a barrier – *‘teachers have many demands placed on them and that integrating movement is another ‘thing’ that they would have to try and ‘fit into’ their curriculum’* [47]. Of greatest concern was the *‘extra time’* required to prepare high-quality PAL lessons [50, 52]. However, practical solutions to minimise the time demands of PAL were *‘keep[ing] it simple’* and using activities that do *‘not take a lot of time to implement and [do not] take a lot of time to set-up’* [26]. As teachers’ activities were repeated, competence developed and, planning and set-up time issues decreased [59].

PAL resources encompassed lesson plans and delivery resources such as number cards or chalk for the playground. Lesson plans were essential to build teachers’ knowledge, this in turn facilitated teachers psychological and physical capability to deliver PAL [19]. The availability of delivery resources reduced barriers which increased the motivation for teachers to deliver PAL [50]. A mixture of DVDs, books, online repositories of PAL ideas and specific delivery equipment are among recommended resources. Having resources and equipment *‘that was ready to hand’* was essential to support frequent delivery [25].

Effective delivery environments and teachers’ ability to use these to deliver PAL was considered key [19]. The most successful integration of PAL was when the *‘entire school was used as a learning space, including halls, playgrounds and green space’* [19]. A teacher’s ability to structure and organise their classroom to promote movement and be creative with the use of the space was related to creating effective delivery environments [24]. For example, *‘one teacher created a ‘city inside of the classroom’ which fosters movement by having supplies, books, and materials located in various locations around the classroom’* [50]. However, a number of studies reflected on how the use of inside space may present issues with safety and noise, which in turn may hinder the use of PAL [54].

The sub-theme finance underpinned most resource issues. It was important for schools to secure external finance for projects or allocate specific resources to support PAL [21, 60]. By doing so, teachers were able to *‘remove barriers to outdoor learning’* by purchasing ready to use equipment [21].

### Whole-of-school approach

While many sub-themes within the whole-of-school approach theme were beyond the control of individual teachers, sub-themes were deemed to positively influence adoption and implementation of PAL. Four sub themes emerged: (i) the role of school culture in implementing PAL, (ii) sustainable implementation of PAL dependent on whole-of-school approach, (iii) senior leaders support for PAL culture, (iv) teamwork and collaboration. The *Social Influences* TDF domain was identified in all sub-themes. *Environmental Context and Resources* was identified in all but one sub-theme; *Social/Professional Role and Identity was* also identified.

Some studies emphasised that the success of PAL *‘may be dependent upon the school culture toward physical activity’* [19]. This may require *‘changing peoples’ attitudes’* [26] toward PAL, and *‘using movement requires a shift in the way teaching and learning is viewed’* [47]. Concerns about early career teachers and *‘how they would look’* when using PAL arose [62]. To build a successful whole-of-school approach and positively influence school culture, required that PAL became everyone’s responsibility and that it be incorporated across all subjects [30, 55].

Most studies emphasised the importance of active buy-in from the school’s senior leadership team and school governors [19–21, 23, 24, 26, 50, 56, 60–62]. Passive involvement was deemed inadequate as teachers preferred head teachers who *‘observed what we were doing and asked how things were going’* [50]. For the program to be effective and sustainable, it was essential that *‘it has to be followed up and monitored and probably fed into performance management’* by head teachers as *‘it just goes, especially if you haven’t got that lead person to keep it ticking over’* [26]. The teachers’ role in supporting the whole-of-school approach was to create communities of practise with colleagues to share and learn. This, in turn, positively influenced social change *‘as everyone does it’* [62]. *‘Timetablers, resource and facility managers’* were considered *‘critical to the logistics of making or preventing change’* [55, 63]. In addition, internal and external communities of practice may all facilitate sustained implementation [20].

### External factors

Two external factors; (i) policy (education and health) and (ii) parents influenced whole-of-school PAL practise. The *Environmental Context and Resources* TDF domain was identified in both sub-themes. *Reinforcement* and *Social Influences* was also identified. Government educational standards placed pressures on schools and heavily influenced PAL delivery. The whole system was referred to as a *‘big pressure cooker’* focussed solely on academic results [19, 21]. Schools and teachers were considered brave if they pursued PAL despite system level barriers [21]. However, in the UK in particular, the Physical Education and School Sport Premium positively influenced schools to move beyond traditional sports to embrace PAL [19]. Parents were a key external influence that positively affect PAL adoption. It was deemed important to educate parents about the benefits of PAL as compared with traditional learning where children sit behind a desk for most of the school day [23].

## Discussion

This is the first meta-synthesis that systematically reviewed and thematically analysed qualitative research evidence regarding themes and sources of implementation behaviours that influence teachers to adopt and implement PAL. Multi-level factors interact to influence teachers’ behaviours [19]. However, too few studies adequately assess these complex interactions. Therefore, we synthesized a broad scope of international studies to assess the perspective of the teachers at the front line of implementing PAL. We reviewed 25 papers -half were published since 2018. They yielded core themes that describe factors that influence teachers’ PAL practise. To better interpret outcomes, we aligned themes (7) and sub-themes (31) with TDF constructs. This theoretical frame positions educators target behaviours to inform PAL teaching training programmes, in future [32].

From themes and sub-themes, a narrative has emerged to describe how a teacher’s capability, opportunity and motivation to deliver PAL could be developed and supported within school systems. Perceived benefits, belief about capabilities, training, delivery and resources describe ‘what needs to be done and when’ for teachers to widely adopt and implement PAL. Whole-school and external themes underline the influence that school systems, policies and stakeholders have on teachers’ PAL behaviour. They also describe how teachers influence these factors. Our synthesis begins to illustrate how inherently complex it is to influence, change and sustain teachers’ PAL behaviours.

### Themes and sources of behaviour underpinning whether teachers adopt and implement PAL

The TDF framework allowed progression from more general and overarching categories of capability, opportunity, and motivation [34] to a more nuanced pattern of behavioural determinants that better reflect the complexity typical of the ecological teaching context to inform teacher training programme development. Theoretically, 10 TDF constructs that influenced teacher behaviour can be used to guide programme design for PAL teacher training (*Knowledge, Skills, Social/Professional Role and Identity, Beliefs about Capabilities, Beliefs about Consequences, Reinforcement, Goals, Environmental Context and Resources, Social influences* and *Emotio*n). TDF stages of development surfaced as important influences on teachers’ behaviours. PAL training programmes must meet the needs of all teachers, not just early adopters or those already motivated to embrace PAL. Four TDF constructs (*Optimism, Intentions, Behavioural Regulation* and *Memory, Attention and Decision Processes*) did not align with our findings. As the PAL teacher training literature advances and more comprehensive PAL programs are developed, the influence of these TDF constructs may emerge.

The seven core themes were not equally represented among the 25 papers we reviewed. Demonstrating the need to educate teachers about the well documented benefits of PAL [14–16], teachers’ beliefs about the benefits of PAL were most widely reported. Beliefs centred on knowledge of educational (21 papers), health (16 papers), enjoyment (15 papers), and improved classroom behaviour (12 papers) benefits that accrue from PAL. Less common were social engagement (5 papers) and communication disparities (3 papers). Aligned with previous literature [21, 23, 27], beliefs about teachers’ personal capabilities was another central theme. Sub-themes centred on trial and error to build skills (10 papers), attitudes toward PAL (8 papers), and confidence in delivering PAL (7 papers). Extending previous understanding, T*rial and error* emerged as a new insight and was described as ‘just have a go’ and learn from the experience [47]. This suggests that teachers need to be open minded and trust the process. This may require training to build relationships and a non-judgemental attitude. Related sub-themes illustrated that a development sequence was followed to build PAL capability. In alignment with the CDC strategies for classroom physical activity in school’s [65], a teacher’s initial mindset early in the process and the importance of PAL and its benefits for pupils, teachers, and schools enabled this. Yet, the analysis suggests the PAL field still must improve the understanding of a teacher’s PAL development journey to improve uptake and implementation. Finally, as previously identified, targeted communication (e.g., websites, practitioner publications) to more widely disseminate findings on the benefits of PAL are needed to facilitate PAL adoption by practitioners [65].

Aligned with current practise guidelines [65], once teachers are recruited to training programmes, the goal is to build teachers’ belief in their ability and delivery skills to implement PAL. Understanding such findings within a behavioural context emphasises the need to address attitude prior to skill development, similar to a stages of change approach to health behaviour. Their cognitive and emotional processes are used initially to build positive attitudes and to motivate [66, 67]. Behavioural processes develop specific actions to support long-term behaviour change. Unique insights drawn from the many studies included in the meta support a process that focuses first on the benefits of PAL to motivate teachers, with a transition to easy to implement lessons and resources to encourage action.

The simpler the action, the less motivation is required to undertake the action [68, 69]. In a school system where many teachers feel they lack time (20 papers), PAL must comprise ideas that are easy to implement. Including PAL in training programs would also build teachers’ confidence [51]. In agreement with current guidelines [65], teachers consistently cited the need for effective delivery environments (17 papers) and PAL delivery resources (15 papers). While the CDC strategy predominantly focusses on classroom-based PAL, many studies within the current review emphasised the need for teachers to embrace teaching in environments beyond the classroom [19, 21]. PAL resources, therefore, must be accessible and easy to use -ideally, each class would have a readily available PAL practical kit.

When teachers were able to manage pupils’ behaviour during PAL lessons, it supported their belief in this personal capability. Similar to previous work, maintaining student focus and classroom control was most consistently cited as a barrier to PAL [26, 65]. Classroom management is a key skill for educational settings, and intimately linked to professional roles and identities [70]. Thus, PAL behaviour management skills and transitions in and out of PAL should be a part of teacher training.

Although simple actions require less motivation, they may result in less satisfying outcomes [68]. To address the notion of differentiation and sustain teachers’ long-term adherence to PAL delivery, novel insights suggested PAL could advance through levels of complexity as teachers’ PAL skills increase and should interest subside. As part of training, more advanced PAL activities could complement a repertoire of simple activities. PAL provided a different learning opportunity that supported students who do not thrive in sedentary classroom settings. A teacher must be able to adapt PAL lessons to meet the needs of all students. It is particularly challenging to meet the specific needs of children with intellectual and physical disabilities; this group is underrepresented in the literature [71–73]. Similarly, PAL might need to be tailored to meet the social and emotional needs of students in or approaching puberty [74]. For example, PAL activities that cause students to sweat may be embarrassing or make students uneasy [75].

To build a teachers’ capability to deliver PAL, it is important that they have access to different learning places and spaces, and that environments can be modified to facilitate movement. Statements such as, ‘it is not safe to be active in classrooms’ or ‘PAL causes too much noise and disturbs other classes’ illustrate barriers that reflect dominant social norms. Extending the previous literature, changing the social norm and building acceptance were key to embedding PAL into school environments [23]. As teachers’ training experience with PAL advances, capacity to fully integrate PAL into curricula increases. Teachers become more able to adapt delivery strategies to each student and different settings, and to adapt and expand PAL content. Implementing and sustaining PAL is linked with integrating PAL into current structures and practices that span school-system levels [19].

Despite its great promise, the PAL development journey, “what to do when,” requires further clarification. Future research might assess the experiences and expertise of other school stakeholders (e.g., principles, parents). With our findings, these data could be used to co-produce a standardised curriculum that underpins development of PAL content, delivery and training that can complement current guidance on embedding PAL in schools [65]. Researchers, with teacher educators, would then be positioned to evaluate the impact of these adapted PAL programs.

Synthesising the results from individual studies has for the first time provided a comprehensive understanding of the role that school context and the broader educational system has on PAL implementation [13, 76]. Building a whole-of-school approach was deemed essential to sustain PAL delivery over the longer term. As with other school-based initiatives [77] and aligned with previous research, actively engaging senior leaders created a positive social norm, and a safe space for teachers to experiment with PAL [60, 65]. For senior leaders to buy-in, PAL outcomes must align with broader system level education goals [23]. For teachers to facilitate leadership buy-in, teachers’ capability to deliver in-house whole-school training via a train-the-trainer approach needs to be enhanced. This may include training materials, lectures and videos that summarise the benefits of PAL and present a range of easy to implement PAL examples. Taking this approach would also influence wider teacher buy-in and help to establish a community of PAL practice.

#### Limitations

We acknowledge some limitations of our study. First, only studies published in English language were reviewed; studies were conducted largely in developed countries. A more inclusive approach that acknowledges differences in school policy and educational philosophy in developing nations would more aptly address key differences in contexts and capacity to deliver PAL. Second, theories or frameworks used to guide the development of the teacher training were not analysed due to the use of thematic synthesis. Within the analysis it became apparent that many studies failed to use theory to guide intervention development and/or structure the analyses. Future studies should seek to address such limitations, while future meta-syntheses may wish to use an alternate analytical approach to appraise the use of theory. Third, despite cultural similarities, different research groups adopted different approaches to PAL. Fourth, our findings are drawn from predominantly researcher-led programmes that are more likely to recruit early adopters. A broader range of teacher views -from early to late adopters- might be incorporated in future. Finally, the analyses, interpretations of the data and alignment of the TDF are influenced by the prior knowledge and experience of the author team. Different authors- due to their own experience and research paradigms may have produced alternative themes, sub themes and TDF alignment.

## Conclusion

In addition to educational benefit, physically active learning (PAL) may also provide a solution to the escalating trend of physical inactivity of children and youth. However, for PAL to be effective and sustain over time, a whole-of-school approach should be considered that embraces learning by teachers and the larger school system. As a first step teachers must receive the training, resources and support to acquire the skills and capabilities to implement and adapt PAL to meet the needs of a diverse range of pupils. The PAL program itself should be adaptable, and progress as teachers’ build their experience and capability; content should be ‘refreshed’ and become more challenging as interest in PAL wanes over time. As a second step, it is imperative to engage all levels of the school community for PAL to be fully integrated into a broader school system. Adequate resources, strong leadership and governance, an engaged ‘activated community and political will are necessary to achieve this [78], and may not currently exist in most schools.

In summary, the meta-synthesis offers novel insights to inform future development of comprehensive PAL teacher training programs and identify gaps that must be filled to effectively implement PAL into schools and sustain implementation over time.

## Supplementary Information

**Additional file 1.**

**Additional file 2.**

## Data Availability

The datasets used and/or analysed during the current study are available from the corresponding author on reasonable request.

## Abbreviations

PAL: Physically active learning
TDF: Theoretical domains framework
