# Supplementary material for: Behaviours that prompt primary school teachers to adopt and implement physically active learning: a meta synthesis of qualitative evidence
Source: Int J Behav Nutr Phys Act. 2021 Nov 20;18:151. doi: 10.1186/s12966-021-01221-9 (PMC8605507; doi:10.1186/s12966-021-01221-9)
Supplement: Supplementary file 1 — Additional file 1. [file 12966_2021_1221_MOESM1_ESM.docx]

**Supplementary Material A Sample Search Strategies**

**EBSCO – (**ERIC, Psych Info, Sport Discus, Academic Search Complete)

( physically active learning OR active learning OR active lessons OR education outside of the classroom OR movement integration OR movement strategies OR physical activity strategies OR classroom physical activity breaks OR classroom breaks OR classroom movement OR move-to-learn OR move to improve OR move-to-improve OR movement and learning OR energisers OR energizers OR class physical activity OR classroom exercise OR physical activity integration)

AND (primary school teachers OR elementary teachers OR headteachers OR teaching assistants OR primary school staff OR teacher trainers )

AND ( facilitators OR barriers OR enablers OR training OR implementation OR feasibility OR delivery OR acceptance OR perception OR adoption OR opinions OR thoughts OR motivation)

**PubMed**

(((“physically active learning” OR “active learning” OR “active lessons” OR “education outside of the classroom” OR “movement strategies” OR “physical activity strategies” OR “classroom physical activity breaks” OR “classroom breaks” OR “classroom movement” OR “move-to-learn” OR “move to improve” OR “move-to-improve” OR “movement and learning” OR “energisers” OR “energizers” OR “class physical activity” OR “classroom exercise” OR “physical activity integration”)) AND (“teachers” OR “headteachers” OR “teaching assistants” OR “primary school staff” OR “teacher trainers”)) AND (“facilitators” OR “barriers” OR “training” OR “implementation” OR “feasibility” OR “delivery” OR “acceptance” OR “perception” OR “adoption” OR “opinions” OR “thoughts” OR “motivation”)

**SCOPUS**

TITLE-ABS-KEY ( ( ( ( "physically active learning" OR "active learning" OR "active lessons" OR "education outside of the classroom" OR "movement strategies" OR "physical activity strategies" OR "classroom physical activity breaks" OR "classroom breaks" OR "classroom movement" OR "move-to-learn" OR "move to improve" OR "move-to-improve" OR "movement and learning" OR "energisers" OR "energizers" OR "class physical activity" OR "classroom exercise" OR "physical activity integration" ) ) AND ( "teachers" OR "headteachers" OR "teaching assistants" OR "primary school staff" OR "teacher trainers" ) ) AND ( "facilitators" OR "barriers" OR "training" OR "implementation" OR "feasibility" OR "delivery" OR "acceptance" OR "perception" OR "adoption" OR "opinions" OR "thoughts" OR "motivation" ) )
