# Supplementary material for: Behaviours that prompt primary school teachers to adopt and implement physically active learning: a meta synthesis of qualitative evidence
Source: Int J Behav Nutr Phys Act. 2021 Nov 20;18:151. doi: 10.1186/s12966-021-01221-9 (PMC8605507; doi:10.1186/s12966-021-01221-9)
Supplement: Supplementary file 2 — Additional file 2. [file 12966_2021_1221_MOESM2_ESM.docx]

Supplementary material B, Table 1: Detailed Thematic synthesis of the 25 papers

| Theme | Sub-theme | Papers | Extracted material | TDF domains |
| --- | --- | --- | --- | --- |
| PAL Benefits | Teachers’ motivation and perceived effects | 1, 2, 5, 7, 8, 9, 13, 14, 15, 19, 20, 21, 23, 24, 25 | Linked to learning  “So, I did a history lesson with primary school kids…. there was one table where I buried artefacts in sand, then they had to solve an Egyptian puzzle with hieroglyphics. It was such a nice lesson; even though it was quite labour intensive to set up, it ran itself perfectly. And every time the music started, they’d move on, so if we could have more lessons like that. (G2)” (Daly-Smith et al., 2020)  Jamie (G4, I) explained how she thought classroom-based physical activities supported the academic work done in class:…if they can do something while they are doing the activity, they actually have it more in their memory because they’re just not sitting there dwelling on it. They’re doing an activity and they have to produce an answer just like that, which is really higher-level thinking.” (Stylianou et al., 2016)  One of the main comments which teachers volunteered during the interview related to how it had prompted renewed reflection on their own teaching style. Questions such as "am I being as creative as possible with my mathematics lessons?" and "are the kids really engaged?" were being asked. (Riley et al., 2017)  Linked to physical activity  “The whole concept made me reflect on the importance of movement for children throughout the day.” (McMullen et al., 2016)  “We are positive to physically active lessons because we have the focus area healthy lifestyle and invest a lot of time in both physical and mental health.” (Skage & Dyrstad 2019)  look answer would be, um, well I have so many standards to address, and I just have to get them all done. But my belief is that I’m here for the better of each child in my classroom...I think physical activity is more important than making sure I can cross one more standard off the list.” (Webster et al., 2017)  “We were positive to the project because the children need to be physically active. Physical activity provides a break to the indoor sitting. I think children are able to achieve more because they are physically active”. Teacher 2 at school 3. (Dyrstad et al., 2018)  “…I think it would be better for them to have more active outlets, and therefore if the school can provide them, that [would] be good.” (Benes et al., 2016) | *Knowledge (#1); Beliefs about Consequences (#6); Reinforcement (#7); Goals (#9)* |
|  | Embracing class diversity | 7, 14, 15, 19, 20, 23 | “I know lots of children that don’t cope very well with being in one classroom all day every day, they find it difficult to sit down but also for children who are more creative, they’ve got more opportunities to show that outdoors, I mean it’s the freedom and the movement and the expression and being able to use their bodies not just their voices and their hand”. (Teacher, School B, Follow up) (Marchant et al., 2019)  “We do have children that have challenging behaviour, but we find they are far more engaged outdoors than indoors”. (Headteacher, School B, Follow up) (Marchant et al., 2019)  “I believe physically active lessons is a fine way to get engagement from the children who do not learn so much from just sitting quietly in the classroom and listening to the teacher.” (Skage & Dyrstad 2018)  “One or two children who have difficulty concentrating, I think they are concentrating a little better and more focused” (Gately et al., 2013)  Eimear writing, “I am a great believer in how movement stimulates the brain, especially for children with difficulties.” (McMullen et al., 2016)  “Some are calmer, but it has the opposite effect on others – who get giddy and over-excited.” (Gately et al., 2013) | *Knowledge (#1), Beliefs about Consequences (#6); Goals (#9)* |
|  | Lack of dissemination of evidence/ communication disparities | 1, 2, 13 | Little research has been passed around to the “people who matter" (Benes, 2016)  “If you haven’t got the evidence to demonstrate that it’s going to work then are you gonna get the buy in? . . . Is there any point trying to parachute in with this if actually the schools don’t buy into it?” (G1) (Daly-Smith et al., 2020)  “if articles from well-respected educational magazines/websites/research were presented to administration, it could be passed down the appropriate chain and become a norm in the classroom.” (Benes et al., 2016) | *Knowledge (#1); Beliefs about Consequences (#6); Social Influences (#12)* |
|  | Pupils’ educational outcomes | 1, 2, 3, 4, 5, 7, 8, 10, 12, 13, 14, 15, 16, 18, 19, 20, 21, 22, 23, 24, 25 | “You learn about the outside world; you notice things about nature you never knew, and you do different topics”. (Pupil, School A, Baseline) (Marchant et al., 2016)  Many students commented that they had found the mathematics easier once they returned to the classroom, as they had "done the maths with [their] bodies”. (Riley et al., 2017)  G1 was keen to stress that such changes “immediately changed the way the children learnt”. (Daly-Smith et al., 2020)  Students used different ways to explain these benefits; "the exercise makes the brain work clearer"; "because your mind has been doing exercise, it kind of gets it ready for mathematics" (Pupils response) (Riley et al., 2017)  “. . if they can do something while they are doing the activity, they actually have it more in their memory because they’re just not sitting there dwelling on it. They’re doing an activity and they have to produce an answer just like that, which is really higher-level thinking.” (Stylianou et al., 2016)  “Teacher 6 said: “The children learn so much from each other when they have to find the answer together, and that’s a good learning experience and has been such a good motivation for me to continue with physically active lessons, since I can see that it works.” (Skage et al., 2020) | *Knowledge (#1) Beliefs about Consequences (#6)* |
|  | Pupils’health | 2, 3, 4, 5, 7, 14, 15, 16, 17, 18, 19, 20, 21, 23, 24, 25 | Several teachers noticed that children with poor motor development and low aerobic fitness improved both their motor skills and fitness. (Dyrstad et al., 2018)  “you sometimes get really out of breath but it’s ok because it’s fun and we’re moving around” (Class 2). Pupil (Norris et al., 2018).  “Is the outcome of active learning to use learning or education to get people more active or is it to help people to learn whilst being active? Which way round is it, or is it both?” (G1) (Daly-Smith et al., 2020)  Other key motivations focused on improving pupil wellbeing and providing more opportunity to be outdoors; “There's far too much time where children aren't playing outside, they aren't walking outside, they aren't just outside, and I think a lot of that, with increasing volumes of children accessing counselling, spending a lot of time on social media, spending a lot of time on Xbox, a lot of time watching TV, they just don't know the impact being outside has on their health and their wellbeing, and I'm really committed to developing pupils' wellbeing”. (Headteacher, School B, Follow up). (Marchant et al., 2019)  Additionally, headteachers accepted physically active lessons based on a decision made to introduce more physical activity and play-based learning for the youngest children. (Skage & Dyrstad, 2019)  ECTs saw their students’ interest and value in physical activity along with student’s positive responses to physical activity as valuable resources and important reasons for incorporating MI into their classrooms. (Webster et al., 2017)  For example, before the intervention started Sorcha wrote that, “I am delighted that the children will get a break from the sheer monotony of sitting in one position for session after session.” (McMullen et al., 2016) | *Knowledge (#1), Beliefs about Consequences (#6); Goals (#9)* |
|  | Pupils’ social engagement and teamwork | 1, 13, 14, 18, 20 | “I would say the biggest thing is engagement, because the kids can’t just sit there and disengage if their main goal is to move around and to engage with each other and with the content.” (Benes)  “I think that like it makes us like learn how to work as a team”. (Pupil, School C, Follow up, Marchant et al., 2019)  Another teacher explained it similarly, “To see positive interaction between children who do everything to solve a challenge or task together.” (Grade 5 teacher) (Lerum et al., 2019)  The group-based activities of the EASY Minds program were found to facilitate more peer learning, support and engagement, providing scope to incorporate students‟ different interests and abilities, in ways that benefitted all students. Indeed, one teacher commented that he had been surprised to see his students “taking ownership” of the program and excited to help set it up, design the activities and so on. (Riley et al., 2017)  “Physically active learning have not only an educational and physical benefit, but it also strengthens the cohesion, cooperation and empathy to the students.” (Grade 5 teacher) (Lerum et al., 2019) | *Knowledge (#1), Beliefs about Consequences (#6)* |
|  | Pupils’ enjoyment and motivation | 1, 3, 4, 5, 7, 9, 13, 14, 15, 16, 18, 20, 22, 24, 25 | “Unique element to the class that students and teachers find enjoyable.” (Benes et al., 2016)  “It’s fun for the children. It (physically active academic lessons) is different than the classroom theory they are so used to, and it provides variation. And it motivates me too, since it gives me the opportunity to get outside. Teacher 1 at school 5. (Dyrstad et al., 2018)  “They like to get involved in anything that doesn’t involve sitting at their desks with a pen!” (Gately et al., 2013)  When considering enjoyment of the students further it also seems that the teachers only chose to repeat the lessons that their students enjoyed, with every reflection that indicated a teacher would try the activity again also including a positively reported reaction from students. (McMullen et al., 2016)  “I liked pretty much everything. It was all entertaining. You could find out something that you didn't know before…then you could do maths with that and figure out your average and just manipulate simple things and just help you learn in a different way.” (Student) (Riley et al., 2017)  Enjoyment interacts with engagement:  ‘It has to be as fun as possible because if people are enjoying what they’re doing, the learning outcomes will come naturally. It’s when they get bored, that you start having to fight people and you’re forcing them to learn again, it becomes a bit more like a classroom’. (Dorling et al., 2020)  Enjoyment ultimately coming from the variation in learning - doing something different  “I think they are excited because [MFT] is a new program that we have never seen before. And more activities that we get to do instead of, you know, the regular things I have been doing... they are going to be really engaged because it’s different”. (Facilitator) Teacher #4, Kindergarten, at School A using MFT (Dugger et al., 2020) | *Knowledge (#1) Beliefs about Consequences (#6); Emotion (#13)* |
|  | Classroom behaviour | 1, 3, 4, 5, 7, 13, 14, 18, 20, 21, 23, 24 | Engagement  “I think of kids being engaged in what they are doing . . . And I see them, basically engaged would be the word that comes to my mind" (Benes et al., 2016)  “I see that I am losing kids now. I’m much more aware of it than I used to be” (Benes et al., 2016)  ‘As far as increasing the engagement and the amount of time on-task, it has definitely had some impact’ (Stephanie, G2) (Stylianou et al., 2016)  (Re-)Focus  “it gives you both a bit of a break as well and it just re-jigs their mind and gets them back on task”. (Norris et al., 2015)  “There were 4 maths lessons that were unusually quiet. The group focused more”; “When the children have finished these sessions they are refreshed and ready to settle down and do work again”; “They are more alert some of them. Engaged and awake. It helps to focus their attention again.” (Gately et al., 2013)  Behaviour Management  A theme that was recurrent throughout the interviews was that of behavioural management. This was conceptualised by teachers as MI potentially having both a positive and a negative impact on classroom behaviour (Routen et al., 2018)  “And it will – it depends with your class. You know, we’ve got quite a few behavioural issues in our school and I imagine in a classroom if you suddenly ask them to jump up and run around, you’ll probably lose about three. And then you’ve got five minutes to kind of get everybody settled back down and – but then I suppose it’s a culture. If you do it every day continuously, they’ll get used to it and it will just become second nature” (Routen et al., 2018)  For example, it was suggested that MI can be used as a tool to ‘re-focus’ pupils when they have been sitting for extended periods and may lose interest/concentration in the present task (Routen et al., 2018)  “If I don’t give them a break, then they check out, they get antsy; I notice more behavioural problems” (Benes et al., 2016) | *Knowledge (#1); Beliefs about Consequences (#6)* |
| Beliefs about own capabilities | Attitudes towards PAL | 11, 12, 15, 18, 19, 22, 23, 25 | “The whole concept made me reflect on the importance of movement for children throughout the day.” (McMullen et al., 2016)  “We wanted to participate in order to further develop our own model, we were afraid of missing out on new knowledge in the area.” (Skage & Dyrstad, 2019)  “An employee survey we recently conducted showed that the teachers are tired of change [in the context of PAL].” (Skage & Dyrstad, 2019)  However, when considering the need for adopting physically active lessons, a majority of the participants reported they already had sufficient activities to increase physical activity in school (e.g., access to sports halls, outdoor school, and physical activity during recess). (Skage & Dyrstad, 2019)  Teachers’ beliefs about the importance of instruction coupled with their lack of knowledge about PA policies and MI specific classroom management proved to be major barriers to their adoption of MI. (Webster et al., 2017)  Teacher 1 said: “I felt that the students got a bit tired of physically active lessons because it was many of the same activities, so I might be a bit tired myself too.” (Skage et al., 2020)  “We already have this activity; we don’t need to do both!”; “I think we do enough physical activity already.” (Gately et al., 2013) | *Belief about Capabilities (#4)* |
|  | Confidence in using PAL | 3, 11, 16, 17, 18, 19, 21 | This creates an issue around confidence in the teacher and their capacity to develop an understanding of effectiveness which leads to a lack of classroom integration and a poor perception of PATL [the PAL intervention] and its implementation strategies. (Mwaanga et al., 2018)  “Confidence in staff, not everyone, even in PE not everybody will go all out, there are some that are reluctant because they lack confidence” (Focus group 1, Teacher 4). (Quarmby et al., 2018)  “Yeah, definitely. It definitely comes down to confidence and their confidence in their own behaviour management ability to get them up and moving because every teacher’s nightmare is, oh my god, I’ve lost control (laughter). So, it tends to be the weaker teachers are less confident” (Routen et al., 2018) | *Skills (#2); Belief about Capabilities (#4)* |
|  | Trial and error | 1, 5, 9, 10, 13, 14, 15, 16, 19, 25 | Challenges... lack of exposure on how to use movement in the classroom (Benes et al., 2016)  “What I found difficult was how to combine physical activity and subjects. There was also a large difference preparing a regular classroom lesson and an outdoor physically active academic lesson…. but gradually such teaching plans (for the active academic lessons) has become easier to prepare.” Teacher 3 at school 1. (Dyrstad et al., 2018)  For example, Chloe (5th grade teacher) mentioned: “I have no idea what I'm doing, so if I don't know how to do it, how are they going to do it? We did kind of fumble through together and figured [it] out.” Nonetheless, her confidence increased over time. She added: “Now I am good so that next year when I have a new class, I will know how to do it. I think my confidence is high. I think since I have done some, I will be more willing to try new ones.” (Goh et al., 2017)  “Right, initially, I thought, “Oh, no!”, because it’s upper school, you tend to focus a lot of written work in class, and obviously foundation phase are used to doing it, so it was a case of, “Oh, where do I start?”, initially. That was my first thought. . . No, I feel more confident now, now that it’s sort of implemented into my teaching. I do feel a bit more confident in preparing outdoor resources”. (Teacher, School B Baseline) (Marchant et al., 2019) | *Skills (#2); Belief about Capabilities (#4)* |
|  | Idea generation | 1, 3, 8 | “So, I just would love to see them move more, but I don’t have a lot of ideas as to what I can do as part of my class to do that.” (Benes et al., 2016)  In addition, teachers wanted more lesson demonstrations by research assistants to gain additional ideas about how to incorporate physical activity into a regular lesson. (Gibson et al., 2008)  ‘Obviously, you come up with your own ideas and what you want to do and what you want to get out of it. But it’s very important to marry that up with the school because that makes your relationship with the school a lot easier’. (Dorling et al., 2020) | *Skills (#2); Social/ Professional Role and Identify (#3); Belief about Capabilities (#4)* |
| PAL training | Importance of PAL training | 2, 9, 14, 16, 18, 20, 25 | Finally, on top of the recognition of Ofsted being essential in PAL implementation, the requirement for PAL to be embedded in ITT programs was emphasized. (Daly-Smith et al., 2020)  “Students who are going into teacher training, they’re getting a minimal amount of PE training. They get 2 h out of the full . . . that sort of needs to be changed so they can have a better understanding.” (G1) (Daly-Smith et al., 2020)  “Lack of training, lack of time to do the job properly….” (Teacher 3) (Mwaanga et al., 2018)  Physical activity in taught lessons as “becoming more the norm” and routinely taught in modern teacher training. (Norris et al., 2015)  Alice concurred: “After the little training that we had, we can just kind of do it, and then just adjusted to what we are doing in class.” (Goh et al., 2017)  “I found the program to be highly engaging; we just thoroughly enjoyed the whole process. From the start the training day, the initial training orientation day with you guys at the University was probably the best day we've had as far as professional development.” (Teacher) (Riley et al., 2017) | *Skills (#2); Beliefs about consequences  (#6)* |
|  | Awareness and knowledge of PAL | 1, 2, 10, 13, 17, 25 | Most other teachers conceived of physical activity as a “break” from learning and had not conceived of physical activity integrated with classroom learning as a possibility. (Graham et al., 2014)  “ASK has opened my eyes to see that it is possible to vary the regular teaching with physical activity.” (Grade 5 teacher) (Lerum et al., 2019) | *Skills (#2)* |
|  | PAL examples, demonstrations and direct experiences | 2, 5, 6, 8, 9, 13, 18, 20, 21, 24 | Besides targeting teacher values/attitudes, it became apparent that future MI programmes may require consideration of individual teaching approaches, both in preferences for particular strategies and overall teaching style. It was suggested that this could be mitigated to a certain extent by providing supporting teaching resources that can be adapted, or through the provision of skills training (Routen et al., 2018)  For example, Jamie revealed: “After we had the training and we got together and we talked about it, I thought this isn't maybe that hard.” (Goh et al., 2017)  “It was nice to know how the body works and how the brain reacts to physical activity. Nevertheless, I still think that the most useful sessions were more practical lectures. How to use different equipment, how to variate physically active learning lessons, etc.” (Grade 5 teacher) (Lerum et al., 2019)  Mr. Street explained, “If there were people available to come out and do a special activity or a special lesson, as long as it is planned within a week, I think it would be a big benefit.” (Egan et al., 2018)  For instance, Christine (G2) shared: ‘I like when you guys show something. I’m probably going to use it more than reading the lessons, because I’ve seen it’ (Stylianou et al., 2016)  In addition, teachers wanted more lesson demonstrations by research assistants to gain additional ideas about how to incorporate physical activity into a regular lesson. (Gibson et al., 2008) | *Skills (#2); Belief about Capabilities (#4); Emotion (#13)* |
|  | Tailored ongoing support | 6, 13, 15, 19, 21, 24 | “The reminders. Again, as teachers we have so much – the standards are changing, and we have testing coming, and we have this special assembly; there is so much going on. Just the ‘Hey, how are you doing? Do you need anything?’ was just kind of like, oh yeah, cause it is in the back of my head.” (Egan et al., 2018)  Ms. Williams described the emails as “a sticky note for your mind.” (Egan et al., 2018)  Besides targeting teacher values/attitudes, it became apparent that future MI programmes may require consideration of individual teaching approaches, both in preferences for particular strategies and overall teaching style. It was suggested that this could be mitigated to a certain extent by providing supporting teaching resources that can be adapted, or through the provision of skills training: (Routen et al., 2018) | *Skills (#2)* |
| PAL Delivery | Planning (lesson integration) | 2, 3, 4, 6, 9, 14, 15, 18, 22, 24, 25 | Paul said: “They did great in the morning, right at 9 o'clock, they will be fantastic. We try to do it at least 2 to 3 times a week… between 10 and 20 min. During winter when we were inside, we did a lot there. And then even when it was raining outside, we would do it.” He added: “There were days when I'd let the kids make up whatever they wanted to, as long as they were moving during that 10 min, it worked really well” (Goh et al., 2017)  She “found it very easy to accommodate the lessons into [her] daily routine.” (McMullen et al., 2016)  Overall, teachers thought that the management techniques taught during the PD sessions were consistent with the management practices they used. For instance, Jessica (G4, I) pointed out some similarities: ‘Make sure to give clear directions, so they know exactly what we’re going to do and what we expect from them’. (Stylianou et al., 2016)  ‘No issues starting but there’s issues stopping. Because some of them don’t have the equippedness [discipline] to stop and we have to work with them a bit more’ (Pamela, G1). (Stylianou et al., 2016)  However, Rachel did explain in the focus group interview that the lessons were, “a great addition to the lessons and that it wasn't something just separate, like that they were part of the lesson.” (McMullen et al., 2016)  When considering scheduling specific statements, Sorcha, who teaches in learning support, wrote that a barrier to implementation was, “Remembering to do them with all the coming and going. I need to schedule them at the beginning of the session.” (McMullen et al., 2016)  Despite this added constraint to MI, the teachers in the high implementers indicated they were able to recognize and capitalize on naturally occurring transition times to integrate new movement opportunities. Mrs. Taylor described her use of MI during transitions: “What I started doing during each rotation, when we would do their work stations throughout the morning, we would do 10 exercises, and I would pick a student that was following expectations, like a student that did a good job focusing on their work, and I would pick [that] student [to lead] and we would do like 10 quick toe touches or whatever … so I looked at how many learning breaks do we have, and I think it is easy to do [MI] during transitions.” (Egan et al., 2018)  “That’s what I liked, it didn’t require a lot of planning time, you could quickly just go to whatever subject area you were covering and quickly find an activity to engage the students in”. (Facilitator Teacher #13, 2nd Grade, at School C using PAAC) (Dugger et al., 2020)  ‘…to like make them all to do that but also focus on actually doing the actual maths as well…we sort of had to change it around to make sure it integrated and linked well with what we wanted to do with both movement games and the education side’. (Dorling et al., 2020) | *Skills (#2)* |
|  | Frequency | 1, 14, 15, 23 | “We’d get bored of it, I wouldn’t do every lesson, I think once or twice a week is enough”. (Pupil, School A, Baseline) (Marchant et al., 2019)  “They use movement in their classrooms, but most do not use it on a regular basis” (Benes et al., 2016)  Rachel, who reported in the focus group interview that she did about five or six lessons per week, said that encouraging teachers to try three lessons “was realistic.” (McMullen et al., 2016)  However, some headteachers reported that individual teachers had started using physically active lessons on their own initiative. (Skage and Dyrstad, 2018) | *Beliefs about Consequences (#6)* |
|  | Intensity | 2, 5, 14, 15, 17 | There was a recognition that the intensity required to deliver health benefits is important. However, this was tempered by an appreciation that it may not be feasible for schools to focus on meeting intensity targets when starting to implement PAL, for example, “to try to contribute to 60 min of MVPA”. (G1) … “The classroom constraint is it’s not a physical environment and if most activities are moderate to vigorously active, you’re not going to be able to learn.” (G3) (Daly-Smith et al.,2020)  She wrote that one specific change she would make would be to, “Showcase the resource in a box of cards easier to use and access,” and that we could, “Mark activities as light, moderate or vigorous.” (McMullen et al., 2016)  “you end up jumping a lot sometimes. it would be good if we could run around more instead” (Class 7) and “it’s ok to move inside sometimes but it would be good if we could have more classes outside as well” (Class 10, E3, Pupil) (Norris et al., 2015) | *Skills (#2); Beliefs about Consequences (#6)* |
|  | Subject Compatibility | 2, 10, 17, 18, 19, 25 | One teacher comment reflected these multiple possible uses well: “I think it’s really adaptable,” she said, and described a few of the ways she could use the mats in her classroom: “It would be cool if they used their hands and feet . . . or if you could hang it on the wall. Or throw a ball on it.” “Or in teams. They have to decide on an answer and run over and click it.”" (Graham et al., 2014)  Somethings just don’t fit. Sometimes you just need to be sat in front of a laptop, you’ve got to be researching or you’ve got to have a text in front of you or reading examples. Comprehension style activities. (Focus group 2, Teacher 1) (Quarmby et al., 2018) | *Skills (#2)* |
|  | Differentiation | 1, 5, 7, 9, 10, 11, 21, 24, 25 | Academic  “It was the same calculations over and over, and I don’t like that very much since we know them by heart. I would like to have more challenging multiplication tasks.” Child (Dyrstad et al., 2018)  “Some of the ones who don’t normally engage in timetables really engage when we do it connected with activity. You’re not tricking them into doing it, you are just engaging them, stimulating them. So, it does work”; “Some children may get the concept better or remember the information better by associating times tables with fun or activity. They can maybe physically see it and remember it, for example, visualise dividing half of the group up for fractions.” (Gately et al., 2013)  Betty (3rd grade teacher) commented: “Some are like, ‘I don't want to do this,’ so they are like faking it and some of them are really lazy in exercise. They didn't want to get up and move.” (Goh et al., 2017)  There were conflicting reports in regard to pupils’ reactions to the dance-based plug-and-play videos. While these activities seemed to work well with younger (K–2) children, teachers of older pupils reported less positive pupil reactions. For instance, Grace (K, I) discussed: ‘They are four and five [years old] and they absolutely love it, they love it. They are all singing and doing the motions and they are very focused’ On the contrary, 3–5 teachers shared comments like ‘Not as much interest from kids with the videos’ (Jessica, G4, SR) (Stylianou et al., 2016)  “in order to understand what they are going to do, we need them to be calmer” (Teacher 7), (Kain et al., 2020) | *Skills (#2); Beliefs about Consequences (#6)* |
|  |  | 9, 10, 19 | Physical  “I know from my son’s point of view, he’s a wheelchair user, that when they do that sort of thing at high school, he’s left at the side, or because it takes him so much time to get into groups of organisation, that he always ends up with the person that no one else wants to work with. So, it’s about ensuring those sorts of physical aspects don’t isolate people" (Focus group 4, Teacher 4) (Quarmby et al., 2018)  Student health and invisible disability were noted barriers. In fact, one participant described it as the most significant barrier to physical activity during the school day. She stated, “This is the biggest one: Asthma. Students with asthma. So many students on inhalers” (Graham et al., 2014) | *Skills (#2)* |
|  |  | 1, 9, 14, 21, 25 | Psychosocial  “If they are very particular about their clothes or their hair depending on how active the movement, it depends on how much jumping around you are doing, I guess” (Benes et al., 2016)  “Yes, because when you’re outside you’re not all sweaty and you like can’t really concentrate that much when you’re like really sweaty but if you’re like outside you’re like nice and cool, so it’ll help you listen better and concentrate better”. (Pupil, School B, Baseline) (Marchant et al., 2019)  For example, Ms. Corsa commented, “sometimes you are trying to get students active and doing things, they’re more concerned with I don’t want to sweat, you know, or I don’t want to look silly, or that’s not cool.” (Webster et al., 2017) | *Skills (#2); Beliefs about Capabilities (#4); Emotion (#13)* |
|  |  | 3, 4, 24, 25 | Age  “You know the Hokey Pokey’s in there too, but I have third graders so they are too cool for that. They weren’t about to do the Hokey Pokey”. (Barrier) Teacher #32, 1st Grade, at School B using Take10 (Dugger et al., 2020)  ‘And the games we were coming up with, we weren’t sure whether they were like age-appropriate but also ability appropriate and whether it would be improving their abilities in school’. (Dorling et al., 2020)  There were conflicting reports in regard to pupils’ reactions to the dance-based plug-and-play videos. While these activities seemed to work well with younger (K–2) children, teachers of older pupils reported less positive pupil reactions. For instance, Grace (K, I) discussed: ‘They are four and five [years old] and they absolutely love it, they love it. They are all singing and doing the motions and they are very focused’ On the contrary, 3–5 teachers shared comments like ‘Not as much interest from kids with the videos’ (Jessica, G4, SR) (Stylianou et al., 2016)  The teachers noted that these behaviours are exhibited more often as the students get older, and the resistance from students has hindered the teachers’ willingness to incorporate more movement opportunities. (Webster et al., 2017) |  |
| Resources | Time | 1, 2, 3, 4, 5, 6, 7, 8, 9, 10, 13, 14, 15, 16, 20, 21, 22, 23, 24, 25 | Teachers have many demands placed on them and that integrating movement is another “thing” that they would have to try and “fit into” their curriculum. (Benes et al., 2016)  Most of the teachers felt that the complexity of the physically active academic lessons was manageable but noted that it was time consuming and difficult to plan and develop physically active academic lessons of high quality. (Dyrstad et al., 2018)  “It’s another planning task to have to do. Planning and prep time is taken up with planning and prep for core subjects. I don’t have the time for extra planning” (Gately et al., 2013)  When considering these daily practicalities and the everyday reality for many teachers outlined in this reflection, suggestions from other participants to “keep it simple”, “not take a lot of time to implement” and “not take a lot of time to set-up” appear meaningful. (Routen et al., 2018)  Teacher 1 said: “When we have done it many times, it does not take more time to prepare a physically active lesson, you will find smart ways to do it.” (Skage et al., 2020) | *Environmental Context and Resources (#11)* |
|  | PAL delivery resources | 2, 4, 5, 9, 10, 12, 13, 14, 15, 16, 18, 19, 21, 24, 25 | Lesson plans  Hence, in order to support teacher’s confidence and competence and provide them with the knowledge of how to incorporate physical activity into their lessons, resources and ready-made schemes of work could be made available. (Daly-Smith et al., 2020)  “It would be have been a lot easier to carry out physically active academic lessons if we had received pre-planned physically active academic lessons, but at the same time it should fit into the part of the subject they (the children) are doing there and then. I realize that this would not have been easy to arrange, but it would have made it easier.” Teacher 1 at school 2. (Dyrstad et al., 2018)  Delivery resources  “… a website link showcasing a few lessons may be beneficial for teachers who don't have the great opportunity for CPD [continuing professional development].” (McMullen et al., 2016)  She wrote, “It would be super if there was a ready-made pack of equipment that could be used for the activities rather than having to search for the equipment … it would be great to have it to hand.” (McMullen et al., 2016)  “I’d also say resources as well, because if all classes are going to be doing active lessons, do we have enough resources for all of the classes?” (FG4, T1) (Quarmby et al., 2018)  Providing resources in a format teachers can easily and quickly access and use. Throughout this project, teachers were provided with several resources, including books, DVDs, links for online resources, and a packet with classroom-based physical activity ideas, which included all the activities demonstrated in the PD sessions (Stylianou et al., 2016)  “It is easier to be motivated (for preparing physically active lessons) when you know that the practical equipment is in place.” Teacher 1 at school 1. (Dyrstad et al., 2018)  “I used the online access and that format was easy to use. Just click on the link”. (Facilitator) Teacher #6, 3rd Grade, at School A using Instant Recess (Dugger et al., 2020) | *Environmental Context and Resources (#11)* |
|  | Delivery environments | 1, 2, 4, 5, 6, 7, 8, 9, 10, 11, 14, 15, 16, 18, 19, 21, 25 | Greater potential was seen if the entire school was used as a learning space, including halls, playgrounds, and green space. (Daly-Smith et al., 2020)  Additionally, Mrs. Williams discussed how she creates a “city inside of the classroom” which fosters movement by having supplies, books, and materials located in various locations around the classroom. The city’s layout prevents traffic jams (e.g., by putting paper in one location, crayons in another, pencils in another, etc.), which also creates more movement opportunities for students, as the children are required to move throughout the classroom to gather supplies.” (Dyrstad et al., 2018)  “Everyone could find a space. We could push desks together to get more floor space. It would just be creative thinking, but it would be manageable.” (Graham et al., 2014)  “There are health and safety implications of doing these activities in a mobile classroom with 34 kids especially with year 6 kids who are big kids”; “The walls are very thin in these classrooms so noisy activities can disrupt the class next door – noise and the shaking of room!” (Gately et al., 2013)  David (5th grade teacher) added: “The problem is space… that would be another hindrance to the program.” (Goh et al., 2017)  “In terms of safety obviously, there are times when I think, “Be careful!” (Teacher 4) (Mwaanga et al., 2018)  “We’re quite limited for space” (Focus group 4, Teacher 1) (Quarmby et al., 2018) | *Skills (#2); Environmental Context and Resources (#11)* |
|  | School finance | 14, 20, 23 | Funding was mentioned by all schools at follow-up. Improved access to funding resulted in resources moving from a barrier to outdoor learning to a facilitator: “Like having ease of access to equipment has been another problem, so we’re trying to change that by we raised some money like I said doing this walk, trying to get equipment that can be accessed by the children and easily and not in a place where, you know, you need a member of staff to go with them”. (Teacher, School A, Follow up) (Marchant et al., 2019)  “An important contributing factor to our acceptance of the School in Motion project (national research project for increased physical activity in lower secondary school) was that it came with extra funds … I think that’s what it takes.” (Skage & Dyrstad, 2019)  Another barrier highlighted by teachers was the clothing required for lessons, having to cancel if some children forgot coats. At follow up, one school had gained financial support, investing it in staffing and outdoor learning specific clothing; “Supported financially, the school have bought waterproofs so that the weather’s not a barrier for the children and yes, they are funding me to continue in September for another year, so yes, very supported”. (Teacher, follow up) (Marchant et al., 2019) | *Environmental Context and Resources (#11)* |
| Whole School Approach | The role of school culture in implementing PAL | 1, 2, 3, 15, 21, 22, 25 | Finally, 1 participant stated that the intensity of delivered PAL may be dependent upon the school culture toward physical activity and the “capability and the confidence of the teachers” (G5) in delivering varying levels of intensity. (Daly-Smith et al., 2020)  “And I think it would just be changing peoples’ attitudes, so maybe providing – you would probably either need to have a member of staff on board that’s very up for it and happy to do some CPD for the rest of the staff. I think it would be a big ask to say to teachers, we want you to teach these practical lessons, teach, I don’t know, science, electricity through practical activities. It’s going to need something to persuade those teachers that the children are going to meet the outcomes that they’re supposed to be doing. You’re going to be, you know be teaching what you’re meant to be teaching” (Routen et al., 2018)  Compounding the lack of training ECTs’ received were concerns about how physical activity “would look” or “how it would take place” (Mr. Chulkas) as well as concerns about classroom management (e.g., calming students down after MI). (Webster et al., 2017)  “Using movement requires a shift in the way teaching and learning is viewed.” (Benes et al., 2016)  “takes a significant shift in the way teachers think about teaching and learning and/or their own teaching practice.” (Benes et al., 2016)  This raises an interesting issue around accepted norms of certain sedentary behaviours and the role of wider society and traditional learning environments in perpetuating these norms. (Routen et al., 2018)  Ms. Golden stated: “For our entire school we do morning movement, which is awesome. We start our day with that, um, and giving students the opportunity to get up and move and allowing it and making it acceptable, I think is definitely an advantage for our school because, again, morning movement, that’s school wide. Everyone does it.” (Webster et al., 2017) | *Social/ Professional Role and Identity (#3); Environmental Context and Resources (#11); Social Influences (#12)* |
|  | Sustainable implementation of PAL dependent on whole-of-school approach | 2, 5, 11, 12, 14, 16, 20, 21, 22 | Teachers commented that they would have liked to see the program extended to other subjects as well (i.e., English), while others thought it should be made available at a whole-school level. (Riley et al., 2017)  A core theme that emerged from the data was the need to take a whole school approach to support teachers in the implementation of movement within the classroom. This was deemed necessary to enable teachers to overcome barriers to MI and facilitate implementation. (Routen et al., 2018) | *Environmental Context and Resources (#11); Social Influences (#12)* |
|  | Senior leaders support for PAL culture | 2, 5, 10, 13, 14, 16, 19, 21, 23, 24, 25 | A core theme that emerged from the data was the need to take a whole school approach to support teachers in the implementation of movement within the classroom. This was deemed necessary to enable teachers to overcome barriers to MI and facilitate implementation. (Routen et al., 2018)  All teachers at the intervention schools felt they received support from the schools’ leaders. However, each principal’s involvement varied from passive to active…. Several teachers also expressed, separately, that follow-up by the school leaders was insufficient. “What I lacked from the school leaders was involvement, that they had observed what we were doing and asked how things were going.” (Teacher 3 at school 1) (Dyrstad et al., 2018)  “It’s essential to have that Head teacher support in everything you do, nothing gets covered unless it has Head teacher approval.” (Focus group 1, Teacher 1) (Quarmby et al., 2018)  “I think the overriding thing that’s in my head is, if you do want to implement something like this, it really has to be a whole school thing. It has to come from the head and the governors. It has to be followed up and monitored and probably fed into performance management. Any big initiative that’s come in or whatever the school’s focusing on, it really has to be implemented and embedded wholly. If it’s just an inset day and it’s not followed up and nobody’s ever asked about it afterwards, it just goes, especially if you haven’t got that lead person to keep it ticking over.” (Routen et al., 2018)  One of the barriers though was the lack of support from administrative and other school staff to implement the program. (Kain et al., 2020) | *Environmental Context and Resources (#11); Social Influences (#12)* |
|  | Teamwork and collaboration | 5, 6, 8, 9, 12, 13, 14, 21, 25 | Similarly, several teachers wanted a forum to share and learn about creative lessons that worked well in other classrooms. Several suggestions included having teachers share lessons at school staff meetings, including examples in a newsletter, or placing creative lessons developed by teachers on a website. (Gibson et al., 2008)  In addition to school wide programs, ECTs expressed how important current collaborative planning and/or how having a support system in place for this purpose is to implementing MI (Webster et al., 2017)  The key findings in this theme centred on the interpersonal processes in the ASK intervention. The opportunity to share and exchange ideas and experiences with researchers and fellow intervention teachers was considered useful and highly appreciated: “We learn a lot from each other when we meet.” (Grade 5 teacher). (Lerum et al., 2019)  Regional gatherings seemed to influence teachers’ agency and enabled them to draw on learning experiences from other teachers when dealing with problems and professional dilemmas. (Lerum et al., 2019)  “There is a need to have all the key players or levers across multiple systems, sitting around the one table at the one time. This includes people like the timetablers, resource and facility managers. These people are often left out but are actually critical to the logistics of making or preventing change.” (Stakeholder 1) (Lander et al., 2020) | *Social/ Professional Role and Identity (#3); Social Influences (#12)* |
| External Factors (outside of teacher level) | Policy (education and health) | 2, 12, 14, 15, 19, 21, 23 | Most discussions supporting this theme emphasized that if PAL did “not directly support academic results then it was questionable whether it would be likely to be supported by the educational setting” (G4), a need for PAL to be compatible with Ofsted criteria was considered and discussed extensively within the workshop. (Daly-Smith et al., 2020)  “You have pressures put on the school from Government, that goes down through the inspectorate, that passes onto the regional consortia, that's passed onto schools, i.e. Headteachers, Governors, Senior Leaders, that's passed onto the teachers, it's passed onto the teaching assistants and it's passed onto the pupils so it's like a big pressure cooker and the whole system, you know, so until there's that change in emphasis right at the top, you know, I think it will always be the brave schools that actually say 'no, this is what I believe in and this is what we'll do'”. (Headteacher, School A, Follow up) (Marchant et al., 2019)  The deputy head teacher noted: “Whatever Ofsted are looking for, that is what every school is doing. So, we had our Ofsted in February and, for the last few years, ever since I’ve been there, my head’s like, “Right, they’re looking for this now, so we’ve got to do some reading,” or they’re looking for SMSC (Spiritual, Moral, Social and Cultural), or they’re looking for multicultural and what we do in that. So whatever Ofsted are looking at, that tends to be what schools do. (Routen et al., 2018)  The School Sports Premium funding also offers schools an opportunity to move beyond the historic “sports” discourse and effectively implement PAL across the whole-school system. (Daly-Smith et al., 2020)  “There needs to be a system level approach to this. Modelled from the top down and bottom up—if we can get it into all aspects of teaching and learning, as research, then I think we will have a chance to really impact teaching.” (Lecturer 2) (Lander et al., 2020) | *Reinforcement (#7); Environmental Context and Resources (#11)* |
|  | Parents | 14, 19, 21, 25 | “I did think we'd have a little bit of resistance at the beginning, because some parents believe children only learn by sitting at a desk, and indeed one grandparent did write on our Twitter account that, "A pity the children weren't sitting at desks writing". . .So, yes, the parents are very positive about the direction that we're going”. (Headteacher, School B, Follow up) (Marchant et al., 2019)  “I think there would be some parents who are thinking how can we move towards being outstanding and suddenly you’re having these, you’re not sitting down having these active lessons” (Focus group 4, Teacher 4) (Quarmby et al., 2018)  “However, the head teacher posted a view contrary to this and suggested that: Parents would think their child should be sitting down and writing for the full hour and working…I think parents see sitting down at tables linked with control, linked with behaviour, linked with attention, all of those things…so sitting is the way that we do it, and it sells the image as well” (P1) (Routen et al., 2018) | *Environmental Context and Resources (#11); Social Influences (#12)* |

PAL, physically active learning. 1 Benes et al., 2016; 2 Daly-Smith et al., 2020; 3 Dorling et al., 2020; 4 Dugger et al., 2020; 5 Dyrstad et al., 2018; 6 Egan et al., 2018; 7 Gately et al., 2013; 8 Gibson et al., 2008; 9 Goh et al., 2017; 10 Graham et al., 2014; 11 Kain et al., 2020; 12 Lander et al., 2020; 13 Lerum et al., 2019; 14 Marchant et al., 2019; 15 McMullen et al., 2016; 16 Mwaanga et al., 2018; 17 Norris et al., 2018; 18 Norris et al., 2015; 19 Quarmby et al., 2018; 20 Riley et al., 2017; 21 Routen et al., 2018; 22 Skage et al., 2020; 23 Skage & Dyrstad 2019; 24 Stylianou et al., 2016; 25 Webster et al., 2017
